# BMAL1 Associates with NOP58 in the Nucleolus and Contributes to Pre-rRNA Processing

**DOI:** 10.1016/j.isci.2020.101151

**Published:** 2020-05-12

**Authors:** Marlene Cervantes, Ignasi Forné, Suman Ranjit, Enrico Gratton, Axel Imhof, Paolo Sassone-Corsi

**Affiliations:** 1 Center for Epigenetics and Metabolism, U1233 INSERM, Department of Biological Chemistry, University of California, Irvine, Irvine, CA 92697, USA; 2 Protein Analysis Unit, Biomedical Center, Ludwig Maximilian University of Munich, Munich 80539, Germany; 3 Laboratory for Fluorescence Dynamics, Department of Biomedical Engineering, University of California, Irvine, Irvine, CA 92697, USA

**Keywords:** Circadian Clock, Nucleolus, Ribosomal RNA, Molecular Mechanism of Gene Regulation

## Abstract

The transcription factor BMAL1 is a core element of the circadian clock that contributes to cyclic control of genes transcribed by RNA polymerase II. By using biochemical cellular fractionation and immunofluorescence analyses we reveal a previously uncharacterized nucleolar localization for BMAL1. We used an unbiased approach to determine the BMAL1 interactome by mass spectrometry and identified NOP58 as a prominent nucleolar interactor. NOP58, a core component of the box C/D small nucleolar ribonucleoprotein complex, associates with Snord118 to control specific pre-ribosomal RNA (pre-rRNA) processing steps. These results suggest a non-canonical role of BMAL1 in ribosomal RNA regulation. Indeed, we show that BMAL1 controls NOP58-associated Snord118 nucleolar levels and cleavage of unique pre-rRNA intermediates. Our findings identify an unsuspected function of BMAL1 in the nucleolus that appears distinct from its canonical role in the circadian clock system.

## Highlights

• BMAL1 displays a circadian-independent localization in the nucleolus
• Bmal1-deficient cells show altered nucleolar morphology
• Interactome proteomics reveals that BMAL1 associates with nucleolar proteins
• BMAL1 appears to play a non-canonical, non-circadian role in pre-rRNA processing

## Graphical Abstract

**Figure:**
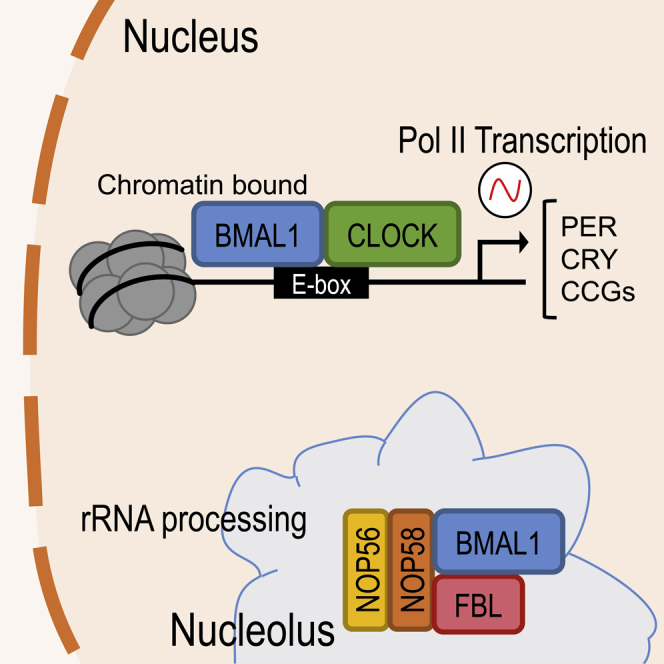

## Introduction

A large array of biological processes follow an approximate 24-h cycle, leading to circadian rhythms controlling physiology, metabolism, and behavior. Circadian rhythms are governed by a molecular clock system that is present in virtually all mammalian cells (Masri and Sassone-Corsi, 2010). The circadian molecular machinery is based on transcription-translation feedback loops to maintain intrinsic intracellular daily rhythms (Eckel-Mahan and Sassone-Corsi, 2013, Zhang and Kay, 2010). Circadian locomotor output cycles kaput (CLOCK) and Brain and muscle ARNT-like 1 (BMAL1) function as transcription factors activating expression of clock-controlled genes (CCGs) by binding E-box motifs at promoters of target genes transcribed by RNA polymerase II (Partch et al., 2014). Among the CCGs transcribed are genes encoding clock protein repressors Period 1-3 (PER1-3) and Cryptochrome 1-2 (CRY1-2), which in turn negatively regulate rhythmic transcription (Green et al., 2008). In addition to transcription, levels of circadian regulation are provided by a variety of additional mechanisms, including epigenetic control and post-translational modifications (PTMs) such as phosphorylation, acetylation, SUMOylation, and ubiquitination of clock proteins that regulate rhythmic activity (Cardone et al., 2005, Feng and Lazar, 2012, Hirayama et al., 2007, Kondratov et al., 2003). Thus, proper circadian control is obtained through a complex system of interplaying regulatory pathways.

Importantly, post-transcriptional modifications of CCG transcripts have also been shown to be critical for rhythmic gene regulation. Alternative splicing, polyadenylation, and non-coding RNAs have all been shown to contribute to control the half-life required for rhythmic turnover of circadian transcripts (Kojima et al., 2011). A unique target of rhythmic post-transcriptional modification is the 18S-E ribosomal RNA (rRNA) (Sinturel et al., 2017), a precursor to an essential component of the small ribosomal subunit (Amaldi et al., 1989, Reuveni et al., 2017). Diurnal polyadenylation of 18S-E results in its degradation, decreased ribosomal biogenesis, and reduced protein translation in a time-of-day-dependent manner (Sinturel et al., 2017).

These observations suggest that circadian clock elements may be implicated in the maturation of transcripts generated not exclusively from Pol II. Indeed, the rRNA precursor (pre-rRNA) is transcribed by RNA polymerase I in the nucleolus as a polycistronic transcript that undergoes post-transcriptional processing to produce three mature rRNAs (18S, 5.8S, and 28S) (Nazar, 2004, Woolford and Baserga, 2013). Nucleolar proteins and small nucleolar RNAs (snoRNAs) form small nucleolar ribonucleoprotein complexes (snoRNPs) that mediate post-transcriptional modifications, pseudouridylation, methylation, and cleavage for rRNA maturation (Boisvert et al., 2007). Specifically, snoRNAs direct snoRNPs to target rRNA sites to facilitate post-transcriptional processing (Boisvert et al., 2007). Intriguingly, bioinformatic analysis of circadian nascent and mature RNA sequencing datasets has recently revealed a time-of-day-dependent expression of a number of snoRNAs (Aitken and Semple, 2017). Although these notions hint at a potential role of clock proteins in the nucleolus, including the nucleolar localization of a splicing variant of Period 2 (PER2S) (Avitabile et al., 2014), a functional role within the sub-nuclear organelle has yet to be explored. We have observed that the core clock protein BMAL1 localizes in the nucleolus and that its ablation results in aberrant nucleolar organization. Importantly, in mouse Neuro 2a (N2a) cells BMAL1 is associated with nucleolar proteins, Nucleolin (NCL) and Nucleolar RNA helicase 2 (DDX21) (Beker et al., 2019). Using cell fractionation and mass spectrometry we reveal that BMAL1 associates with NOP58, a core component of the box C/D small nucleolar ribonucleoprotein complex. The association is validated by a number of approaches, including fluorescence lifetime microscopy and fluorescence resonance energy transfer (FLIM-FRET). Finally, we show that BMAL1 is required to maintain Snord118-containing snoRNP levels and plays a previously unappreciated role in pre-rRNA processing.

## Results

### Nucleolar Localization of the Circadian Clock Protein BMAL1

**Figure 1:**
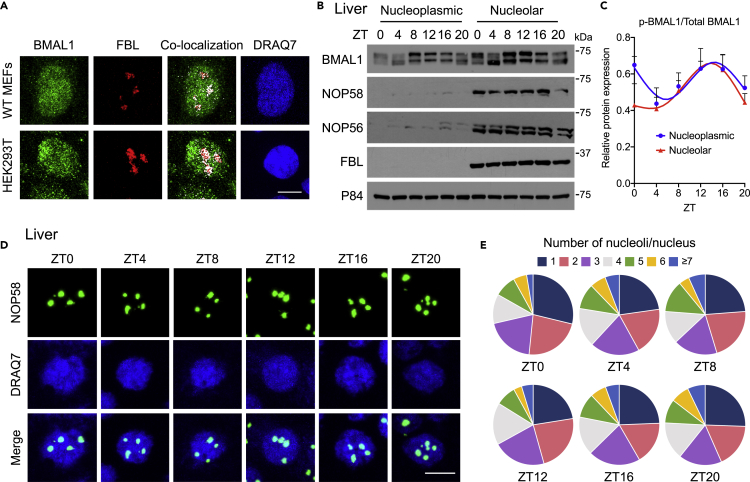
BMAL1 Localization in Circadian-Independent Nucleoli (A) Representative images of endogenous BMAL1 (green) and FBL (red) immunofluorescence in WT MEFs and HEK293T cells with DRAQ7 nuclear stain (blue) and co-localized pixels (white). Scale bar, 10 μm. A total of 10 distinct fields of view were imaged/cell type. (B) Western blot analysis of endogenous BMAL1 and nucleolar proteins, NOP58, NOP56, and FBL, in nucleoplasmic and nucleolar fractions prepared from WT mouse liver tissues harvested at ZT0, 4, 8, 12, 16, 20. p84 was used as a loading control. (C) Quantification of phosphorylated BMAL1 (p-BMAL1) as a ratio of total BMAL1 from (B). Data are presented as mean + SEM. N = 5 biological replicates/time point/group, no significant differences by two-way ANOVA with Sidak's multiple comparisons test. (D) Representative images of endogenous NOP58 (green) immunofluorescence in WT mouse livers harvested at ZT0, 4, 8, 12, 16, 20 with DRAQ7 nuclear stain (blue). Scale bar, 10 μm. A total of 6–9 distinct fields of view were imaged/time point. (E) Pie charts representing the percentage of nuclei displaying the indicated number of nucleoli in WT mouse livers from (D) identified by NOP58 at ZT0, 4, 8, 12, 16, 20. N = 3 biological replicates/time point, a total of 2,493 cells were counted.

Although post-transcriptional polyadenylation of 18S-E rRNA is diurnal (Sinturel et al., 2017), little is known about the contribution of clock proteins in the nucleolus, the specific location of rRNA synthesis and processing (Nazar, 2004, Woolford and Baserga, 2013). Visualization of BMAL1 by immunofluorescence in wild-type mouse embryonic fibroblasts (WT MEFs) and human embryonic kidney 293T cells (HEK293T) illustrated that endogenous BMAL1 is not excluded from the nucleolus and rather co-localizes with the nucleolar protein, Fibrillarin (FBL) (Figure 1A). Moreover, we performed biochemical isolation of nucleoli using liver tissues harvested from WT mice at six time points throughout the circadian cycle, at zeitgeber times (ZT) 0, 4, 8, 12, 16, 20 (Figures 1B and S1A). Analysis of endogenous BMAL1 revealed its nucleolar localization at all circadian time points. Consistent with the total protein analyses (Figure S1B), FBL, Nucleolar protein 58 (NOP58), and Nucleolar protein 56 (NOP56) levels in the nucleolus were virtually constant along the circadian cycle (Figure 1B).

Circadian function is directly linked to changes in BMAL1 phosphorylation state (Hirano et al., 2016). Notably, daily changes in phosphorylated-BMAL1(p-BMAL1), conventionally visualized as slower migrating bands (Tamaru et al., 2003, Yoshitane et al., 2009), do not affect its nucleolar localization (Figures 1B and 1C). Moreover, as BMAL1 has also been shown to be rhythmically acetylated and is strongly correlated to its circadian transcriptional activity (Hirayama et al., 2007), we tested whether acetylation alters BMAL1 localization to the nucleolus. We ectopically expressed mutants of BMAL1 for Lysine at position 538, originally shown to be the site of acetylation (AcBMAL1K538) (Hirayama et al., 2007). HEK293T cells transiently co-transfected with GFP-NOP58 and WT Myc-BMAL1, Myc-BMAL1K538R preventing acetylation, or the acetylation mimic Myc-BMAL1K538Q all show localization in the nucleolus despite distinct BMAL1 acetylation statuses (Figure S2). Thus, these PTMs do not appear to be involved in BMAL1 nucleolar localization and confirm that BMAL1 does not undergo rhythmic nucleolar translocation.

Furthermore, circadian analyses of NOP58 by immunofluorescence show that the number of nucleoli per nucleus remains constant throughout the 24-h cycle in mouse livers (Figures 1D and 1E). Together, these observations show that the nucleolar features are consistent throughout the daily cycle and suggest BMAL1 is constitutively present in the nucleolus.

### Clock-Dependent Nucleolar Morphological Rearrangements

**Figure 2:**
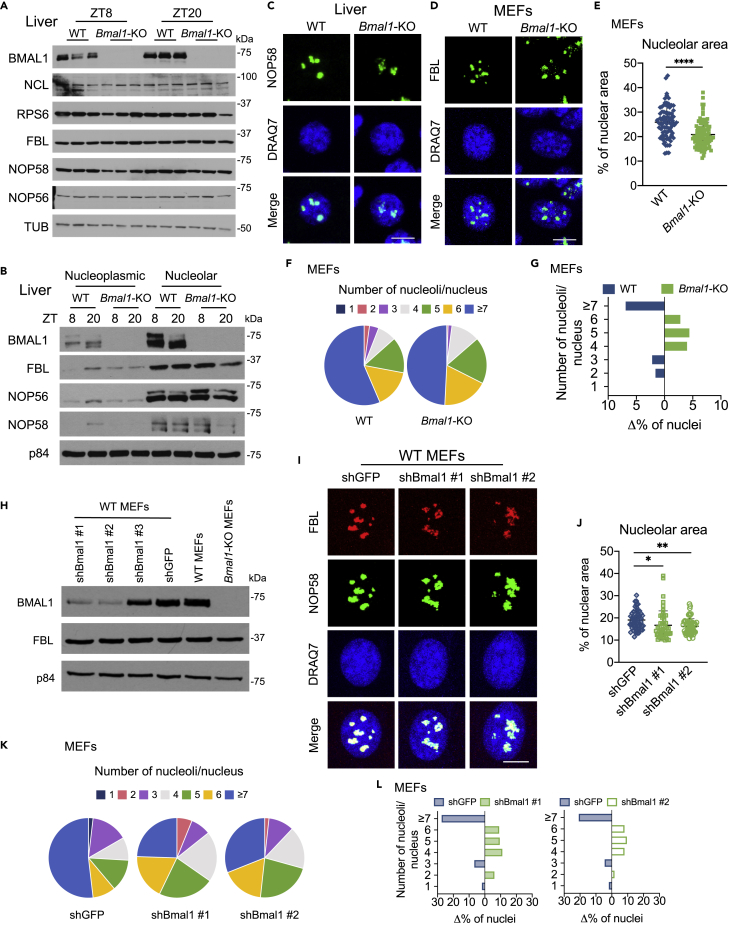
*Bmal1*-Deficient Cells Show Altered Nucleolar Structure (A) Western blot analyses of whole-cell extracts prepared from WT and *Bmal1*-KO mouse livers collected at ZT8 and ZT20 for ribosome biogenesis proteins, Nucleolin (NCL), Ribosomal Protein S6 (RPS6), FBL, NOP58, and NOP56. Tubulin (TUB) is used as a loading control. (B) Western blot analysis of endogenous BMAL1, FBL, NOP56, and NOP58 in nucleoplasmic and nucleolar fractions prepared from WT and *Bmal1*-KO mouse liver tissues harvested at ZT8 and ZT20. p84 was used as a loading control. (C) Representative images of endogenous NOP58 (green) immunofluorescence in WT and *Bmal1*-KO mouse livers harvested at ZT8 with DRAQ7 nuclear stain (blue). Scale bar, 10 μm. A total of 10 distinct fields of view were imaged/genotype. (D) Representative images of endogenous FBL (green) immunofluorescence in WT and *Bmal1*-KO MEFs with DRAQ7 nuclear stain (blue). Scale bar, 10 μm. A total of 14 distinct fields of view were imaged/genotype. (E–G) Nucleolar measurements. (E) Total nucleolar area measured as the percent of nuclear area with FBL signal in WT and *Bmal1*-KO MEFs. Individual cells are plotted. N = 3 biological replicates/group, ∗∗∗∗p < 0.0001 by unpaired t test with Welch's correction. (F) Pie charts representing the percentage of nuclei displaying the indicated number of nucleoli in WT and *Bmal1*-KO MEFs from (D) identified by FBL. (G) Delta percentage of WT and *Bmal1*-KO MEFs that display the indicated number of nucleoli per nucleus. A total of 353 cells were counted. (H) Western blot analyses of BMAL1 and FBL in whole-cell lysates prepared from *Bmal1*-KO MEFs, and WT MEFs stably expressing shGFP, shBmal1 #1, shBmal1 #2, or shBmal1 #3. p84 was used as a loading control. (I) Representative images of endogenous FBL (red) and NOP58 (green) immunofluorescence in WT MEFs stably expressing shGFP, shBmal1 #1, or shBmal1 #2 with DRAQ7 nuclear stain (blue). Scale bar, 10 μm. A total of 8 distinct fields of view were imaged/condition. (J–L) Nucleolar measurements. (J) Total nucleolar area measured as the percent of nuclear area with FBL and NOP58 signal in WT MEFs stably expressing shGFP, shBmal1 #1, or shBmal1 #2. Individual cells are plotted. N = 4 technical replicates/group, ∗p < 0.05 and ∗∗p < 0.01 by one-way ANOVA with Tukey's multiple comparisons test. (K) Pie charts representing the percentage of nuclei displaying the indicated number of nucleoli in MEFs from (I) identified by FBL and NOP58. (L) Delta percentage of WT MEFs stably expressing shGFP or shBmal1 #1 (left), and shGFP or shBmal1 #2 (right) that display the indicated number of nucleoli per nucleus. A total of 218 cells were counted.

To determine the significance of BMAL1 in the nucleolus we analyzed the expression of nucleolar proteins in livers from WT and *Bmal1*-null (*Bmal1*-KO) mice at ZT8 and ZT20. Although BMAL1 ablation does not alter the total levels of ribosome biogenesis factors or within the nucleolar fractions (Figures 2A and 2B), it has a substantial impact on nucleolar structure. Indeed, immunofluorescence of NOP58 and FBL shows that loss of BMAL1 leads to alterations in nucleolar size and the number of nucleoli per nucleus, both in mouse liver and MEFs (Figures 2C and 2D). The total nucleolar area in *Bmal1*-KO MEFs appears smaller (Figure 2E). Likewise, the number of nucleoli in *Bmal1*-KO MEFs is reduced when compared with WT MEFs, which display greater than 5% more cells with seven or more nucleoli per nucleus (Figures 2F and 2G). These results suggest that BMAL1 influences the configuration of nucleoli. As BMAL1 and CLOCK are dimer partners, we tested dominant-negative *Clock*-mutant MEFs containing a deletion in the *Clock* gene at Exon 19 (*Clock*Δ19). These cells provide (1) an additional model with a disrupted clock and (2) the potential of CLOCK to influence nucleolar structures similar to its partner, BMAL1. Indeed, the *Clock*Δ19 MEFs display smaller and fewer nucleoli (Figures S3A–S3D). Moreover, similarly to *Bmal1*-KO MEFs, *Clock*Δ19 MEFs maintain comparable levels of NOP58 and FBL (Figure S3E). Importantly, these results were corroborated in WT MEFs stably expressing short hairpin RNAs efficiently knocking down BMAL1 levels (Figures 2H and S3F). Immunofluorescence imaging of FBL and NOP58 in these cells show reduced nucleolar area and lower number of nucleoli compared with WT MEFs expressing control shGFP (Figures 2I–2L). Again, a reduction in the number of nucleoli was observed without disruption in the level of nucleolar protein FBL (Figure 2H). Our findings show that BMAL1 perturbation in *in vitro* and *in vivo* models leads to nucleolar-protein rearrangements, reflected by changes in nucleolar structure, and allude to dysfunctional nucleoli (Nemeth and Grummt, 2018, Scheer and Hock, 1999).

### Altered Pre-ribosomal RNA Processing in *Bmal1*-KO

**Figure 3:**
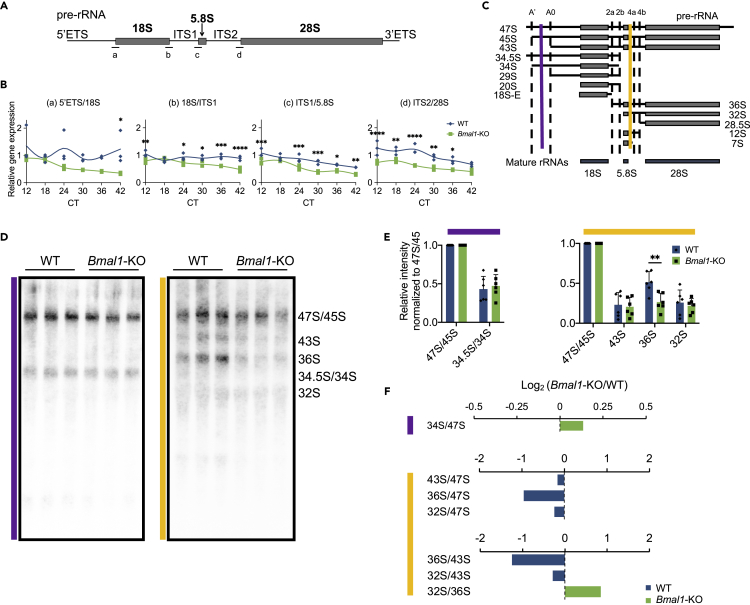
Pre-ribosomal RNA Processing Is Altered in the Absence of BMAL1 (A) Schematic representation of the mammalian pre-rRNA transcript (ETS, external transcribed spacer; ITS, internal transcribed spacer). a–d indicate primer sequence positions designed for reverse transcriptase-quantitative PCR (RT-qPCR) analyses. (B) Pre-rRNA expression profiles at indicated regions a–d from (A) in WT and *Bmal1*-KO MEFs harvested at circadian times (CT) 12, 18, 24, 30, 36, 42 h post-synchronization. Gene expression normalized to 18S rRNA. Individual data points are plotted. N = 3 technical replicates/time point/group, ∗p < 0.05, ∗∗p < 0.01, ∗∗∗p < 0.001, and ∗∗∗∗p < 0.0001 by two-way ANOVA with Sidak's multiple comparisons test. (C) Schematic representation of mouse pre-rRNA cleavage intermediates (laterally labeled) as depicted by Henras et al. (2015). Dotted lines delineate labeled cleavage sites (top). Vertical colored lines (purple and gold) show regions of hybridization by the Northern probes designed by Lapik et al. (2004). (D) Northern analyses of pre-rRNA intermediates in WT and *Bmal1*-KO MEFs. Colored lines correspond to colored probes from (C); intermediates identified are labeled on the right. Equal amount of total RNA was loaded for analysis. (E) Quantification of each intermediate from (D) normalized to 47S/45S. Colored lines label graphs corresponding to colored probes from (C). Data are presented as mean + SD. N = 6 biological replicates/group, ∗∗p < 0.01 by two-way ANOVA with Sidak's multiple comparisons test. (F) Ratio Analysis of Multiple Precursors (RAMP) quantification of each intermediate from (D). Colored lines label graphs corresponding to colored probes from (C). Data are presented as the Log_2_ (*Bmal1*-KO/WT) of the mean ratio of each cleavage pair. N = 6 biological replicates/genotype.

The nucleolus is the site of pre-rRNA transcription and maturation, producing three of four rRNAs, 18S, 5.8S, and 28S (Boisvert et al., 2007). To investigate the functional contribution of BMAL1 in the nucleolus, we examined the expression of the polycistronic pre-rRNA transcripts in a circadian manner in WT and *Bmal1*-KO MEFs post-synchronization by dexamethasone at circadian times (CT) 12, 18, 24, 30, 36, and 42. To distinguish pre-rRNA from mature rRNA, pre-rRNA expression was measured at four distinct regions spanning both rRNA and external transcribed spacer (ETS) or internal transcribed spacer (ITS) sequences (Figure 3A). While pre-rRNA expression does not undergo circadian oscillations (Figure 3B), BMAL1 ablation leads to reduced levels of pre-rRNA. During rRNA processing, sequential cleavage of pre-rRNA diverges into two pathways, one containing the upstream 18S intermediates; the other, the downstream 5.8S and 28S intermediates (Henras et al., 2015) (see also Figure 3C). Therefore, to discriminate the different cleavage site intermediates indistinguishable by PCR (RT-qPCR), levels of pre-rRNA were examined by Northern analyses using probes discerning pre-rRNA cleavage at different stages (Figures 3C–3F and S4A–S4D) (Lapik et al., 2004). Indeed, the specific cleavage intermediate, 36S pre-rRNA, was significantly less abundant in the absence of BMAL1 compared with the WT MEFs (Figure 3E). Similarly, Ratio Analysis of Multiple Precursors (RAMP) (Wang et al., 2014) shows hybridization patterns that reflect the importance of BMAL1 in efficient pre-rRNA processing (Figure 3F). These results indicate that BMAL1 is likely involved in proper processing of 36S, the 3′-end of pre-rRNA containing 5.8S and 28S rRNAs.

### Endogenous BMAL1 Nucleolar Interactome

**Figure 4:**
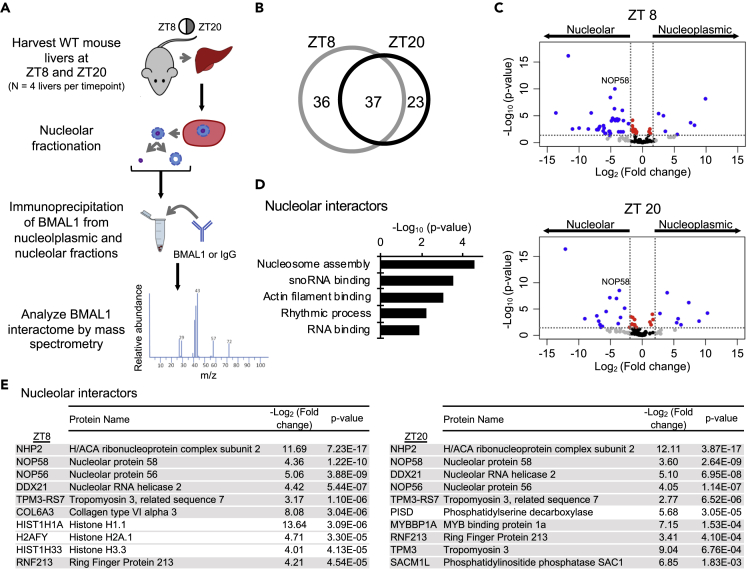
Identification of the BMAL1 Nucleolar Interactome (A) Schematic representation of the experimental design for the nucleolar BMAL1 interactome analysis. Mass spectrometric analysis was performed on N = 4 biological replicates/time point. (B) Venn diagram of the number of total significant BMAL1-interacting proteins at ZT8 and ZT20 compared with IgG control. Significant interactors were identified when having 2-fold differences between nucleoplasmic and IgG or nucleolar and IgG, respectively, with p < 0.05 as determined by two-way ANOVA adjusted for multiple comparisons. (C) Volcano plots of nucleoplasmic versus nucleolar BMAL1 interactors at ZT8 and ZT20. Significant interactors were identified when having 4-fold differences (Log_2_ (Fold change) nucleoplasmic/nucleolar > |2| and p < 0.05 (blue) as determined by two-way ANOVA adjusted for multiple comparisons). (D) Gene Ontology (GO) analysis of the significant nucleolar BMAL1-interacting proteins (Fisher exact p value denotes annotation significance). (E) Top 10 significant nucleolar BMAL1 interactors listed with p value and -Log_2_ (Fold change) of the comparison between nucleoplasmic/nucleolar. Proteins highlighted in gray appear at both time points.

Next, we sought to identify whether BMAL1 associates with nucleolar proteins. Notably, in N2a cells, BMAL1-associated proteins include nucleolar proteins, NCL and DDX21 (Beker et al., 2019). Thus, we explored the link between BMAL1 and nucleolus-derived proteins by taking an unbiased approach to examine the nucleolar BMAL1 interactome. Endogenous BMAL1 was used for a native co-immunoprecipitation (co-IP) from nucleoplasmic and nucleolar fractions of mouse livers harvested at ZT8 and ZT20. The eluate was subsequently analyzed quantitatively by mass spectrometry (Figure 4A). A comparison of the two time points revealed a 50%–60% overlap of proteins identified at both time points (Figures 4B and S5A). Each of the fractions analyzed contained a group of exclusive interactors and a subgroup of proteins interacting in both, nucleoplasmic and nucleolar fractions (Figures 4C and S5B). Importantly, no uniquely associated nucleoplasmic interactors were identified in the nucleolar fractions, an indication of highly enriched nucleoli. Moreover, when comparing the enrichment of BMAL1-interacting proteins in the nucleoplasmic and nucleolar fractions, we identified the subset of BMAL1 interactors present predominantly in the nucleolar compartment by using a cutoff of 4-fold enrichment (-Log_2_ (Fold change) nucleoplasmic/nucleolar ≥2) and a p value < 0.05 (Figure 4C). We applied Gene Ontology (GO) analysis to these BMAL1-nucleolar interactors and identified essential nucleolar-related terms: snoRNA binding and RNA binding (Figure 4D). Prominent among the proteins identified in the nucleolus were box C/D proteins (NOP58, NOP56, and FBL), box H/ACA protein (NHP2), and previously identified nucleolar RNA helicase, DDX21 (Beker et al., 2019) (Figures 4E and S5C). Of note, SIRT7, a prominent nucleolar deacetylase involved in pre-rRNA processing (Chen et al., 2016, Iyer-Bierhoff et al., 2018, Sirri et al., 2019), does not interact with BMAL1 (Figure S5D). Collectively, these data illustrate that the BMAL1 interactome contains highly specific nucleolar interactors.

### BMAL1 Interaction with Nucleolar Protein NOP58

**Figure 5:**
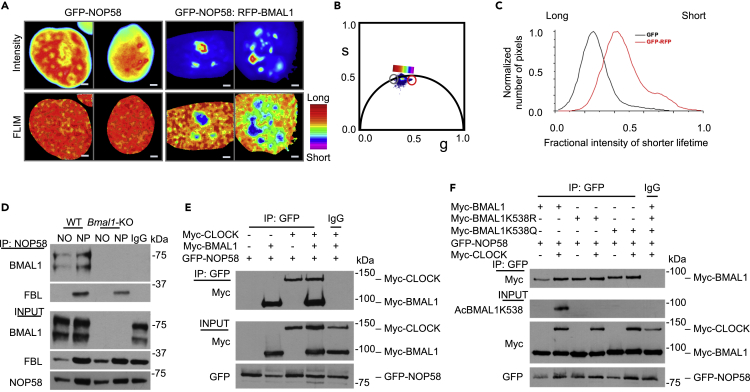
BMAL1 Interacts with Nucleolar Protein NOP58 (A) Representative images of HEK239T cells transfected with GFP-NOP58 and RFP-BMAL1 as indicated and displayed as auto-scaled fractional intensity images (top) and phasor-mapped FLIM images (bottom) color mapped according to the color scheme distribution (left) depicting a range from long to shorter lifetime. Scale bars, 2 μm. (B) Phasor plot of FLIM images in (A) where red is representative of long lifetime and purple is representative of shorter lifetime and more FRETting. Gray circle denotes GFP-NOP58 cells, and red circle denotes GFP-NOP58:RFP-BMAL1 cells. (C) A cumulative histogram showing the number of pixels plotted against fractional intensity of the shorter lifetime in GFP-NOP58 only (black) and GFP-NOP58:RFP-BMAL1 (red). (D) Endogenous co-immunoprecipitation analyses were performed in nucleoplasmic (NP) and nucleolar (NO) fractions prepared from WT or *Bmal1*-KO mouse livers. Immunoprecipitation (IP) was performed using BMAL1 or rabbit IgG followed by western blot analyses of BMAL1, FBL, and NOP58 as specified. (E) Co-immunoprecipitation analyses performed in HEK293T cells co-transfected with GFP-NOP58, Myc-BMAL1, and Myc-CLOCK as indicated. Immunoprecipitation (IP) was performed using GFP or rabbit IgG followed by western blot analysis of Myc and GFP as specified. (F) Co-immunoprecipitation analyses were performed in HEK293T cells co-transfected with GFP-NOP58 and Myc-BMAL1, Myc-BMAL1K538R, or Myc-BMAL1K538Q mutants. Immunoprecipitation (IP) was performed using GFP or rabbit IgG followed by western blot analyses of Myc, GFP, and AcBMAL1K538 as specified.

Our mass spectrometric analyses identified NOP58, which forms snoRNP complexes in the nucleolus that are critical for pre-rRNA processing (Filipowicz and Pogacic, 2002), as a highly significant nucleolar BMAL1-interacting protein. To visualize the interaction between NOP58 and BMAL1, we carried out a combination of FLIM-FRET live cell imaging. For FRET pairing, HEK293T cells were transiently transfected with GFP-NOP58, used as the donor molecule, and RFP-BMAL1, the acceptor molecule (Figure S6A). When in close proximity, energy transfer from the donor molecule to the acceptor reduces the lifetime of the donor (Digman et al., 2008). Visualization by FLIM using a continuous color scheme on the phasor map from red to purple denotes a shift of the phasor points toward shorter lifetime. Accordingly, phasor-mapped FLIM analysis of the GFP-NOP58:RFP-BMAL1 or GFP-NOP58-only cells reveal blue puncta unique to the GFP-NOP58:RFP-BMAL1 cells illustrating energy transfer and an interaction (Figures 5A and 5B). Moreover, histograms of the normalized number of pixels of the fractional intensity of lower lifetime from the GFP-NOP58-only or GFP-NOP58:RFP-BMAL1 cells plotted cumulatively (Figure 5C), and individually (Figure S6B), compare the complexity within each cell and evaluate differences between cells. Thus, the greater intensity shift toward shorter lifetime displayed by the puncta in GFP-NOP58:RFP-BMAL1 cells indicate areas where the two proteins are in close contact. Consistent with the mass spectrometric data, these results validate the BMAL1 and NOP58 association and demonstrate that the interaction is occurring in the nucleoli of live cells.

Next, we tested whether BMAL1 is required for the association between the box C/D proteins, NOP58 and FBL. We performed co-IP of endogenous NOP58 in nucleoli fractionated from livers of WT and *Bmal1*-KO mice (Figure 5D). BMAL1 ablation did not alter the NOP58-FBL association.

Noteworthy, CLOCK was identified as a BMAL1 interactor by mass spectrometry in both the nucleoplasmic and nucleolar fractions (Figure S5B). To establish whether BMAL1 requires its canonical partner to interact with NOP58 we examined the interaction of GFP-NOP58 with Myc-BMAL1 in the presence and absence of Myc-CLOCK (Figure 5E). These results indicate that heterodimerization with CLOCK is not necessary for interaction of BMAL1 with NOP58. This finding was confirmed by using truncated forms of BMAL1 showing that both PER-ARNT-SIM (PAS) domains, involved in CLOCK dimerization (Huang et al., 2012), are not required for NOP58 interaction (Figure S7). Therefore, the BMAL1 and NOP58 association is likely independent of CLOCK.

Finally, to interrogate whether BMAL1 acetylation, a signature of circadian activity (Hirayama et al., 2007), influences its interaction with NOP58, we transiently transfected the mutated Myc-BMAL1K538 constructs described earlier with GFP-NOP58 for co-IP in HEK293T cells (Figure 5F). NOP58 associates with BMAL1, AcBMAL1K538, BMAL1K538R, and BMAL1K538Q equally, indicating that BMAL1 acetylation is not essential for the interaction. Taken together, these results show that the NOP58-BMAL1 association is resilient to circadian disruption.

### BMAL1 Is Involved in Snord118 Recruitment to Box C/D snoRNP

**Figure 6:**
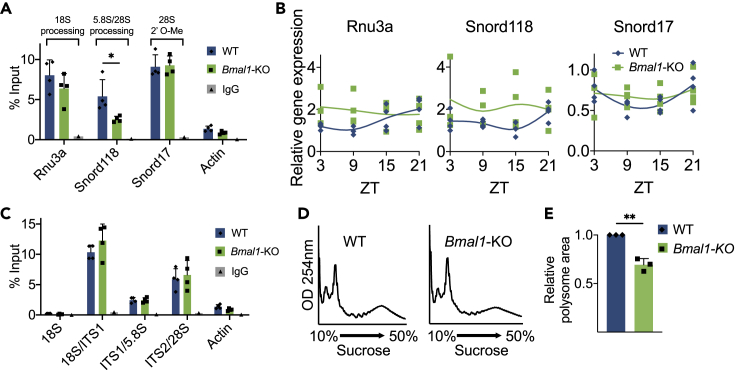
BMAL1 Links snoRNA Recruitment to Box C/D snoRNP (A) RNA immunoprecipitation (RIP) using NOP58 or rabbit IgG in nucleolar fractions isolated from WT and *Bmal1*-KO mouse livers followed by qPCR analyses of snoRNAs: Rnu3a, Snord118, and Snord17. *Actin* mRNA was used as a negative control. Data are presented as mean + SD. N = 4 biological replicates/group, ∗p < 0.05 by two-way ANOVA with Tukey's multiple comparisons test. (B) Rnu3, Snord118, and Snord17 snoRNAs expression profiles in WT and *Bmal1*-KO mouse livers harvested at ZT3, 9, 15, 21. Gene expression was normalized to 18S rRNA. Individual data points are plotted. N = 4 biological replicates/time point/group; no significant differences by two-way ANOVA with Sidak's multiple comparisons test. (C) RIP using NOP58 or rabbit IgG in nucleolar fractions from WT and *Bmal1*-KO mouse livers followed by qPCR analysis for 18S rRNA, 18S/ITS1, ITS1/5.8S, and ITS2/28S pre-RNAs. *Actin* mRNA was used as a negative control. Data are presented as mean + SD. N = 4 biological replicates/group, no significant differences by two-way ANOVA with Tukey's multiple comparisons test. (D) Polysome profiles of WT and *Bmal1*-KO MEFs. (E) Quantification of the area under the polysome curve from (D). Data are presented as mean + SD. N = 3 biological replicates/group, ∗∗p < 0.01 by unpaired t test.

Pre-rRNA processing relies on two dominant classes of snoRNP complexes, box C/D protein containing- and box H/ACA protein containing-snoRNPs (Watkins and Bohnsack, 2012). In combination with distinctive box C/D family snoRNAs, NOP58, and the core box C/D proteins FBL, NOP56, and SNU13, form unique snoRNPs with one of two functions directed by the specialized snoRNA within each complex (Caffarelli et al., 1998). Specifically, nucleolar box C/D snoRNPs carrying box C/D snoRNAs such as MBI-43 (Snord17) or MBII-135 (Snord65) carry out rRNA *2′ O-*methylation; snoRNAs U3 (Rnu3a), U8 (Snord118), or U22 (Snord22) guide pre-rRNA processing (Caffarelli et al., 1998, Huttenhofer et al., 2001, Lestrade and Weber, 2006, Watkins and Bohnsack, 2012). Thus, we sought to assess whether BMAL1 impacts the RNA composition of box C/D snoRNP complexes by performing RNA immunoprecipitation followed by quantitative PCR (RIP-qPCR). NOP58 was immunoprecipitated from isolated nucleoli derived from livers of WT and *Bmal1*-KO mice. Analysis of the bound RNA for Rnu3a, a snoRNA known to guide 18S processing (Beltrame and Tollervey, 1995, Kass et al., 1990), displayed no changes in recruitment to the snoRNP complex in *Bmal1*-KO mice (Figure 6A). Similarly, Snord17 predicted to guide 28S *2′ O*-methylation (Huttenhofer et al., 2001) was equally recruited to the snoRNP upon BMAL1 ablation. In contrast, Snord118, which binds 28S during 5.8S/28S processing (Peculis, 1997), showed significant reduction in association with NOP58 despite being equally expressed in *Bmal1*-KO mice (Figure 6B). Moreover, recruitment of the snoRNP complex to the different modified regions, pre-rRNAs 18S/ITS1, ITS1/5.8S, and ITS2/28S, was not altered in *Bmal1*-KO mice (Figure 6C). Thus, BMAL1 appears to direct the specific association of NOP58 with Snord118, a snoRNA required for 5.8S and 28S rRNA maturation.

Mature rRNAs, in association with ribosomal proteins, constitute the fundamental components of a functional ribosome (Amaldi et al., 1989, Reuveni et al., 2017). Misregulation or interruption of rRNA processing significantly alters ribosome biogenesis and function (Meskauskas et al., 2003, Sloan et al., 2017). Therefore, we assessed whether ribosome biogenesis was perturbed in the absence of BMAL1 by analyzing polysome accumulation (Figure 6D). A comparison of polysome profiles in WT and *Bmal1*-KO MEFs shows a significantly dampened polysome accumulation, indicating that BMAL1 ablation leads to fewer assembled ribosomes compared with WT MEFs (Figure 6E). Accordingly, BMAL1 contributes to snoRNP complex assembly and proper pre-rRNA maturation with a downstream effect on the formation of ribosomes.

## Discussion

The proteins that constitute the circadian core clock machinery are classically identified as transcriptional regulators (Eckel-Mahan and Sassone-Corsi, 2013, Masri and Sassone-Corsi, 2010, Partch et al., 2014, Zhang and Kay, 2010). It has also been suggested that, to maintain cellular homeostasis, they may play additional roles in protein translation, PTMs, and protein translocation (Cardone et al., 2005, Feng and Lazar, 2012, Hirayama et al., 2007, Kondratov et al., 2003). Here we report that the clock protein, BMAL1, is a component of the nucleolus. It is conceivable that other circadian clock proteins may also localize to the nucleolus, as it appears to be the case for the PER2S splice variant (Avitabile et al., 2014), and as further hinted by the presence of CLOCK in our mass spectrometric analysis. Although additional investigations are needed to promote this possibility, it should be stressed that other Pol II-associated transcription factors have also been found to localize in the nucleolus. Indeed, notable examples of transcription factors localized in the nucleolus include the TBP-related factor TRF2, the factor and insulator CTCF, EGR1, C/EBPa, and the virus-encoded protein MEQ (Ali et al., 2008, Arabi et al., 2005, Grandori et al., 2005, Kieffer-Kwon et al., 2004, Liu et al., 1997, Muller et al., 2010, Ponti et al., 2014, Torrano et al., 2006). Although the possible function of these factors in the nucleolus remains undefined, it is likely to be unconventional with respect to their canonical role in transcriptional regulation of protein-coding genes. In this respect, our findings are relevant as we identify a direct role of BMAL1 in the nucleolus that seems independent of Pol II-mediated transcriptional regulation.

We have demonstrated that BMAL1 ablation in distinct models including mouse livers and MEFs elicits nucleolar rearrangement by decreasing the size and number of nucleoli per nucleus. Consequently, given that nucleolar structure and function are tightly correlated (Nemeth and Grummt, 2018, Scheer and Hock, 1999), we hypothesized that BMAL1 plays a role in nucleolar function. However, to address whether BMAL1 is directly involved in the reorganization of the nucleolus will require in-depth structural studies and analysis by phase-separation compartmentalization (Frottin et al., 2019).

To elucidate the consequences of the disruption to the nucleolar structure induced by BMAL1 ablation, we identified the endogenous nucleolar BMAL1-binding partners by mass spectrometry from native, uncrosslinked protein-protein interactions. Notably, BMAL1 associates with box C/D snoRNP proteins, NOP58 and FBL, which are directly involved in pre-rRNA processing (Filipowicz and Pogacic, 2002). Also, BMAL1-related proteins NCL and DDX21, are nucleolar proteins critical for ribosomal biogenesis (Beker et al., 2019). Indeed, confirmation of these interactions *in vivo* and *in vitro* supports a scenario that establishes a causal link between BMAL1 interaction with the snoRNP proteins and impaired pre-rRNA processing of the 3′-end containing 5.8S and 28S rRNAs observed in the BMAL1-null cells. Our study suggests a molecular mechanism by which the function exerted by BMAL1 likely involves the specific interaction with a unique snoRNA, Snord118. Loss-of-function experiments have shown that Snord118 directs 5.8S/28S processing (Peculis, 1997). Although the detailed molecular mechanism by which BMAL1 exerts its function within the nucleolus will need further exploration, our results establish the remarkable example of a Pol II transcription factor operating at a completely different level of transcript regulation. Furthermore, it is plausible that the downstream effects of this regulation are complementary to the promotion of protein synthesis mediated by BMAL1 phosphorylation to control global protein translation (Lipton et al., 2015). In conclusion, our findings position BMAL1 in company with unconventional RNA polymerase II-associated transcription factors previously found to have nucleolar localization (Ali et al., 2008, Arabi et al., 2005, Grandori et al., 2005, Kieffer-Kwon et al., 2004, Liu et al., 1997, Muller et al., 2010, Ponti et al., 2014, Torrano et al., 2006). Importantly, this non-canonical function of BMAL1 in the nucleolus appears to operate in a circadian-independent manner.

### Limitations of the Study

BMAL1 is found to be localized in the nucleolus. Yet, the exact ratio of BMAL1 molecules at chromatin versus the nucleolus remains undetermined. We acknowledge that nucleolar fractions are merely a representation of enriched nucleoli, displaying augmented levels of BMAL1 specifically in the nucleolus with the limitation of not being a full representation of the nucleolar amount with respect to the total nuclear extract. Further studies are needed to investigate this ratio and whether it might be modified in the presence of additional circadian-dependent regulators.

### Resource Availability

#### Lead Contact

Further information and requests for resources and reactions should be directed to and fulfilled by the Lead Contact, Paolo Sassone-Corsi (psc@uci.edu).

#### Materials Availability

All unique/stable materials and models generated from this study are available from the Lead Contact with a completed Materials Transfer Agreement.

#### Data and Code Availability

The MS data generated in this study are available via ProteomeXchange: PXD018946.

## Methods

All methods can be found in the accompanying Transparent Methods supplemental file.

## Supplemental Information

Document S1. Transparent Methods and Figures S1–S7
