# Supplementary material for: BMAL1 Associates with NOP58 in the Nucleolus and Contributes to Pre-rRNA Processing
Source: iScience. 2020 May 12;23(6):101151. doi: 10.1016/j.isci.2020.101151 (PMC7256328; doi:10.1016/j.isci.2020.101151)
Supplement: Document S1. Transparent Methods and Figures S1–S7 [file mmc1.pdf]

iScience, Volume *23*

Supplemental Information

BMAL1 Associates with NOP58 in the Nucleolus and Contributes to Pre-rRNA Processing

Marlene Cervantes, Ignasi Forné, Suman Ranjit, Enrico Gratton, Axel Imhof, and Paolo Sassone-Corsi

SUPPLEMENTAL FIGURES

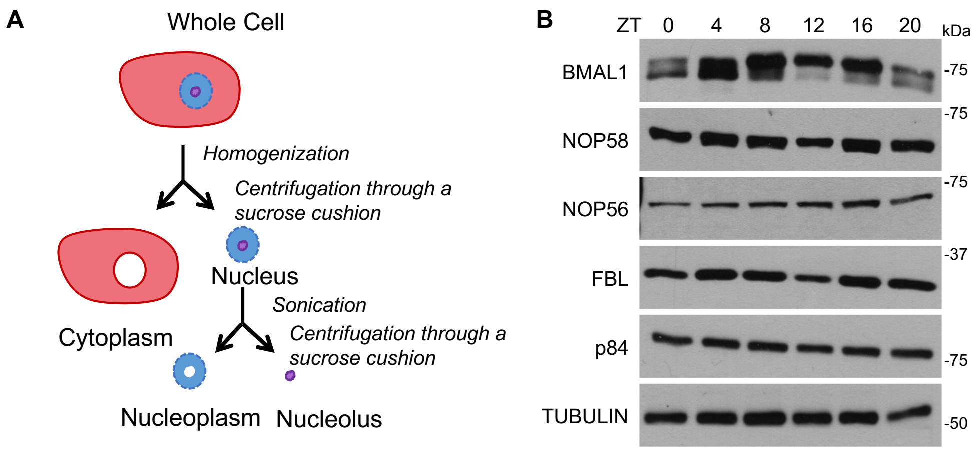

Figure S1 Related to Figure 1. Nucleolar proteins are not circadian

(A) Schematic representation of the experimental design used for nucleolar fractionation.

(B) Western blot analyses of whole cell lysates prepared from WT mouse livers collected at ZT0, 4, 8, 12, 16, 20, blotted for BMAL1, NOP58, NOP56, and FBL. p84 and TUBULIN were used as loading controls.

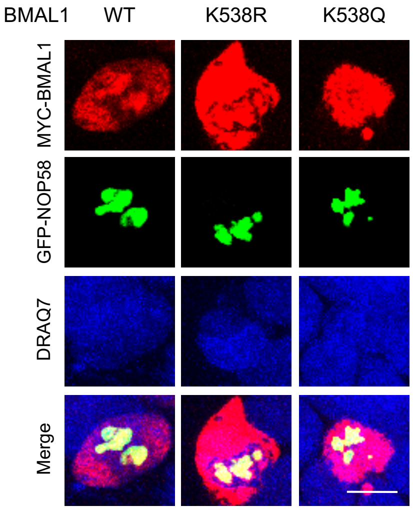

Figure S2 Related to Figure 1. BMAL1 nucleolar localization is acetylation-independent

Representative images of HEK293T cells co-transfected with GFP-NOP58 and Myc-BMAL1 (WT), Myc-BMAL1K538R (K538R), or Myc-BMAL1K538Q (K538Q) mutants. MYC was used to visualize the Myc-BMAL1 variants (red) with DRAQ7 nuclear stain (blue). Scale bar, 10µm. A total of 9 distinct fields of view were imaged/condition.

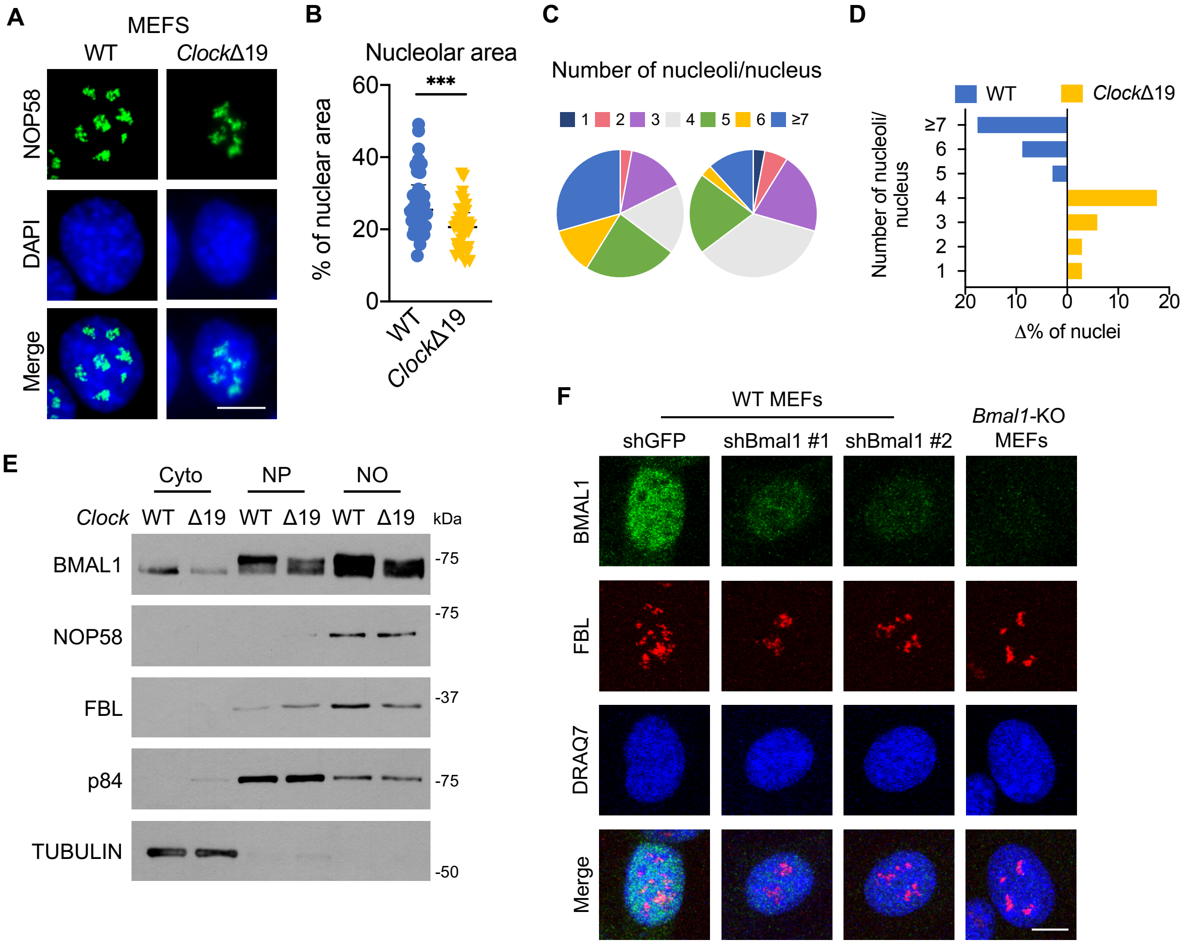

Figure S3 Related to Figure 2. The BMAL1 effect on nucleolar structure

(A) Representative images of endogenous NOP58 (green) in WT and *Clock*Δ19 MEFs with DAPI nuclear stain (blue). Scale bar, 10µm. A total of 5 distinct fields of view were imaged/condition.

(B-D) Number of nucleoli. (B) Total nucleolar area measured as the percent of nuclear area with NOP58 signal in WT and *Clock*Δ19 MEFs. Individual cells are plotted. N=3 technical replicates/group, ***p<0.001 by unpaired t test with Welch's correction. (C) Pie charts representing the percentage of nuclei displaying the indicated number of nucleoli in WT and *Clock*Δ19 MEFs from (A) identified by NOP58. (D) Delta percentage of WT and *Clock*Δ19 MEFs that display the indicated number for nucleoli per nucleus. A total of 68 cells were counted.

(E) Western blot analyses of BMAL1, NOP58, and FBL in cytoplasmic (Cyto), nucleoplasmic (NP), and nucleolar (NO) fractions prepared from WT and *Clock*Δ19 MEFs. p84 and TUBULIN were used as loading controls.

(F) Representative images of endogenous BMAL1 (green) and FBL (red) in *Bmal1*-KO and WT MEFs stably expressing shGFP, shBmal1 #1, or shBmal1 #2 with DRAQ7 nuclear stain (blue). Scale bar, 10μm. A total of 5 distinct fields of view were imaged/condition.

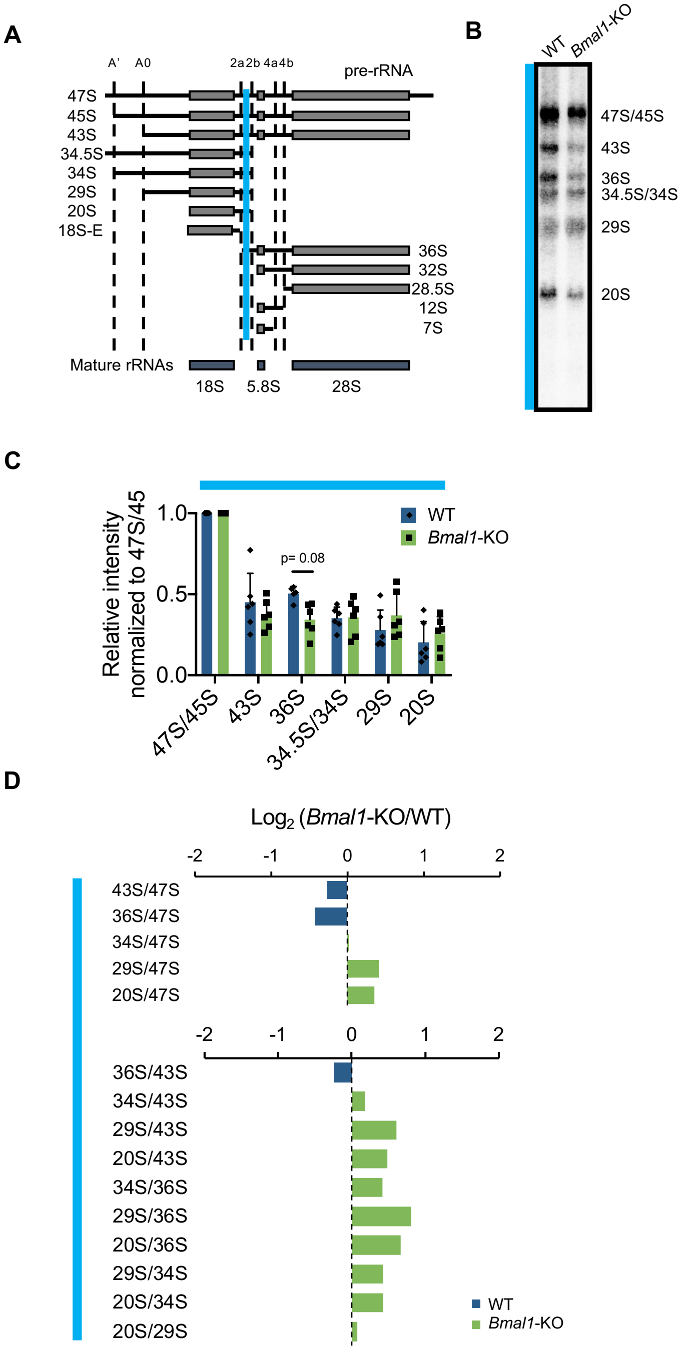

Figure S4 Related to Figure 3. Reduced pre-rRNA processing in *Bmal1*-KO MEFs

(A) Schematic representation of mouse pre-rRNA cleavage intermediates (laterally labeled) as depicted by (Henras et al., 2015). Dotted lines delineate labeled cleavages sites (top). Vertical

colored line (blue) shows regions of hybridization by the Northern probe designed by (Lapik et al., 2004).

(B) Northern blot analysis of pre-rRNA intermediates in WT and *Bmal1*-KO MEFs. Colored line (blue) corresponds to colored probe from (A), intermediates identified are labeled on the right.

(C) Quantification of each intermediate from (B) normalized to 47S/45S. Colored line corresponds to colored probe from (A). Data are presented as mean + SD. N=6 biological replicates/group, significance tested by two-way ANOVA with Sidak's multiple comparisons test.

(D) Ratio analysis of multiple precursors (RAMP) quantification of each intermediate from (B). Data are presented as the Log_2 (*Bmal1*-KO/WT) of the mean ratio of each cleavage pair. N=6 biological replicates/genotype.

A All significant nucleolar interactors

ZT8 only

Protein	ZT8 Log2 (Fold Change)	ZT8 p-value	ZT20 Log2 (Fold Change)	ZT20 p-value
HIST1H1B	8.85	1E-02	3.81	2E-01
COL6A1	8.15	8E-04	1.74	4E-01
BGN	7.71	4E-03	2.22	3E-01
CISD3	7.26	2E-02	2.56	4E-01
HMGN1	7.11	1E-02	0.00	1E+00
TINAGL1	6.99	4E-03	1.55	5E-01
ELOVL2	6.84	3E-02	5.51	7E-02
ABCB10	6.73	1E-03	3.45	7E-02
HIST1H1A	6.67	4E-03	-4.74	3E-02
HMGN2	6.27	4E-02	0.42	9E-01
COL6A2	6.05	2E-03	1.56	4E-01
CBX3	5.81	2E-02	0.00	1E+00
HIST2H2AB	5.53	2E-02	0.00	1E+00
HIST3H2BA	5.26	8E-03	0.00	1E+00
BANF1	5.21	4E-02	0.00	1E+00
PARP1	5.18	8E-03	0.00	1E+00
DIDO1	5.05	2E-02	2.15	3E-01
DCTN2	4.39	2E-02	2.37	2E-01
DCN	3.76	1E-02	0.00	1E+00
H2AFV	3.67	1E-03	-0.45	6E-01
MYH4	3.35	4E-02	0.00	1E+00
HIST2H2AC	3.27	1E-03	0.12	9E-01
MYH10	3.06	1E-02	0.00	1E+00
H2AFY	3.03	2E-03	-0.64	5E-01
HIST1H2B	2.92	3E-03	-0.12	9E-01
H1F0	2.75	2E-03	-0.65	4E-01
HIST1H33	2.65	2E-03	-0.56	5E-01
HIST1H1E	2.54	2E-03	-0.47	5E-01
MYH9	1.92	1E-03	0.61	2E-01
HIST1H1C	1.92	2E-02	-0.91	3E-01
HIST1H4	1.81	7E-03	-0.18	8E-01
MYO1C	1.32	3E-02	0.68	2E-01
EPPK1	1.26	3E-02	0.04	9E-01
TGM2	1.18	8E-03	0.12	8E-01
TPM3-RS7	1.15	2E-02	0.91	5E-02
MYL6	1.13	4E-02	0.53	3E-01

Both

Protein	ZT8 Log2 (Fold Change)	ZT8 p-value	ZT20 Log2 (Fold Change)	ZT20 p-value
ATP5SL	14.61	5E-07	14.62	5E-07
ARNTL	12.73	2E-08	12.09	5E-08
RAB3IP	12.42	1E-06	12.45	1E-06
LPL	10.12	1E-06	9.51	3E-06
CLOCK	10.12	1E-05	9.47	3E-05
PIGX	10.01	6E-04	7.42	7E-03
LRCH3	9.74	5E-20	9.59	6E-20
ABHD16A	9.72	5E-09	9.95	4E-09
ASGR2	8.95	5E-04	9.86	2E-04
ILKAP	8.33	1E-05	8.74	6E-06
PISD	8.12	3E-07	7.40	1E-06
COL6A3	8.08	3E-06	3.78	6E-03
MYL12B	8.02	5E-03	8.37	4E-03
RALGAPA1	7.30	4E-13	7.97	8E-14
CRACR2B	6.19	2E-04	6.55	1E-04
SACM1L	6.14	4E-03	6.23	4E-03
TMEM102	6.02	4E-03	6.03	4E-03
NIFK	5.91	2E-02	6.65	9E-03
FAM213A	5.87	2E-02	5.10	5E-02
PKP2	5.84	1E-02	4.92	3E-02
RNF213	5.00	6E-06	5.22	3E-06
PPA2	4.66	2E-02	5.76	4E-03
SSFA2	4.43	1E-02	4.35	1E-02
DOCK7	3.18	4E-05	3.39	2E-05
FLNA	3.04	3E-02	3.34	2E-02
GPX4	2.53	5E-03	3.11	1E-03
ZBTB20	2.32	5E-04	2.11	1E-03
HVM58	2.11	3E-04	2.97	5E-06
SLC39A7	2.00	2E-04	1.66	9E-04
NHP2	1.88	1E-04	2.30	1E-05
ASGR1	1.69	6E-06	1.84	2E-06
NOP58	1.34	8E-04	1.35	8E-04
SEC31B	1.27	3E-03	1.67	2E-04
C1RA	1.22	4E-03	1.42	1E-03
FLNB	1.14	2E-02	1.90	6E-04
C1SA	1.07	2E-02	1.29	7E-03
NOP56	1.01	5E-02	1.32	1E-02

ZT20 only

Protein	ZT8 Log2 (Fold Change)	ZT8 p-value	ZT20 Log2 (Fold Change)	ZT20 p-value
TRY4	7.09	7E-02	8.91	2E-02
TOMM22	2.68	4E-01	7.81	3E-02
P2RX7	4.36	8E-02	7.04	9E-03
ACTA2	4.36	7E-02	6.74	9E-03
HACD3	2.14	5E-01	6.59	3E-02
CAPZB	4.25	1E-01	6.11	4E-02
PZP	2.65	1E-01	5.13	4E-03
SLCO1B2	2.24	3E-01	4.89	5E-02
TPM3	4.60	5E-02	4.85	4E-02
MYBBP1A	2.91	7E-02	4.25	1E-02
IGK-V19-17	2.57	2E-01	3.89	4E-02
SRPRB	2.02	2E-01	2.86	5E-02
HVM10	-0.08	9E-01	1.78	2E-02
NCL	0.98	5E-03	1.63	5E-05
DDX21	1.13	7E-02	1.60	1E-02
C1QA	0.62	2E-01	1.59	2E-03
KV6A4	0.69	2E-01	1.35	1E-02
FMO1	0.62	2E-01	1.22	2E-02
COX4I1	0.14	8E-01	1.22	4E-02
NDUFS5	0.88	1E-01	1.20	4E-02
CAPZA2	0.88	2E-02	1.16	2E-03
FARSB	0.77	4E-02	1.11	5E-03
CYP2C37	0.79	1E-01	1.07	5E-02

B Top 10 Common nucleoplasmic and nucleolar interactors

ZT8

Nucleoplasmic Protein	Log2 (Fold change)	p-value	Nucleolar Protein	Log2 (Fold change)	p-value
LRCH3	9.68	5E-20	LRCH3	9.74	5E-20
ABHD16A	9.41	9E-09	ABHD16A	9.72	5E-09
BMAL1	12.07	5E-08	BMAL1	12.73	2E-08
RAB3IP	12.77	8E-07	ATP5SL	14.61	5E-07
ATP5SL	13.85	1E-06	RAB3IP	12.42	1E-06
LPL	9.72	2E-06	LPL	10.12	1E-06
PISD	6.98	2E-06	ILKAP	8.33	1E-05
ILKAP	8.29	1E-05	CLOCK	10.12	1E-05
CLOCK	9.60	2E-05	ASGR2	8.95	5E-04
ASGR2	9.42	3E-04	PIGX	10.01	6E-04

ZT20

Nucleoplasmic Protein	Log2 (Fold change)	p-value	Nucleolar Protein	Log2 (Fold change)	p-value
LRCH3	9.17	1E-19	LRCH3	9.59	6E-20
ABHD16A	9.37	9E-09	ABHD16A	9.95	4E-09
BMAL1	11.56	9E-08	BMAL1	12.09	5E-08
ATP5SL	14.05	8E-07	ATP5SL	14.62	5E-07
RAB3IP	12.66	9E-07	RAB3IP	12.45	1E-06
LPL	9.72	2E-06	LPL	9.51	3E-06
CLOCK	9.13	4E-05	ILKAP	8.74	6E-06
ASGR2	10.31	1E-04	CLOCK	9.47	3E-05
NDUFA1	9.20	3E-02	ASGR2	9.86	2E-04
TRY4	8.60	3E-02	TRY4	8.91	2E-02

C

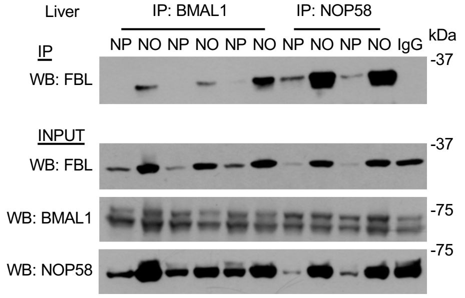

D

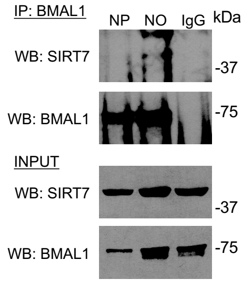

Figure S5 Related to Figure 4. Endogenous BMAL1 nucleoplasmic and nucleolar associations

(A) All BMAL1 interactors identified at ZT8, ZT20 or both, listed with p-value and Log_2 (Fold change) of the comparison between nucleolar and IgG.

(B) Top 10 common BMAL1 interactors in nucleoplasmic and nucleolar fractions at ZT8 and ZT20, listed with p-value and Log_2 (Fold change) of the comparison between nucleoplasmic or nucleolar and IgG. BMAL1 and CLOCK are highlighted in gray.

(C) Endogenous co-immunoprecipitation experiments were performed in nucleoplasmic (NP) and nucleolar (NO) fractions prepared from WT mouse livers. Immunoprecipitation (IP) was performed using BMAL1, NOP58 or Rabbit IgG followed by Western blot (WB) analyses of BMAL1, FBL, and NOP58 as specified.

(D) Endogenous co-immunoprecipitation experiments were performed in nucleoplasmic (NP) and nucleolar (NO) fractions prepared from WT mouse livers. Immunoprecipitation (IP) was performed using BMAL1 or Rabbit IgG followed by Western blot (WB) analyses of BMAL1 and SIRT7 as specified.

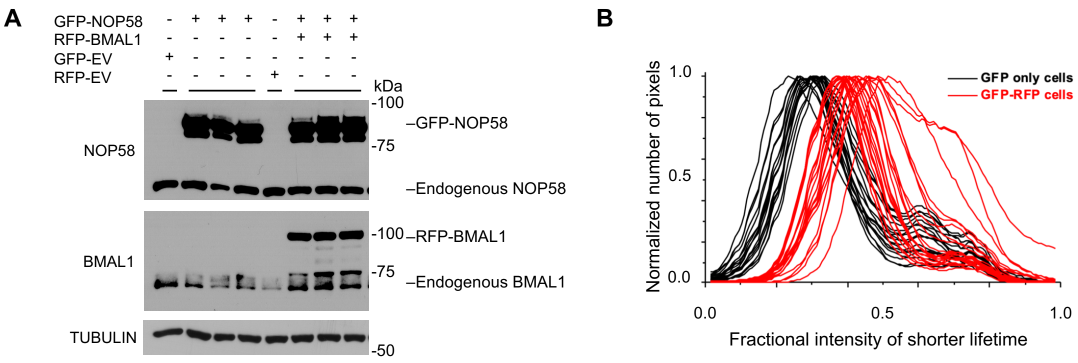

Figure S6 Related to Figure 5. Intranuclear BMAL1-NOP58 interaction

(A) Western blot analyses of HEK293T cells transfected with GFP-NOP58, RFP-BMAL1, or the respective empty vectors (EV) as indicated, showing the levels of exogenous GFP- or RFP-tagged proteins compared the respective endogenous protein. TUBULIN was used as a loading control.

(B) Histogram (individual cells plotted) showing the number of pixels plotted against fractional intensity of the shorter lifetime in GFP-NOP58 only (black) and GFP-NOP58:RFP-BMAL1 (red) cells.

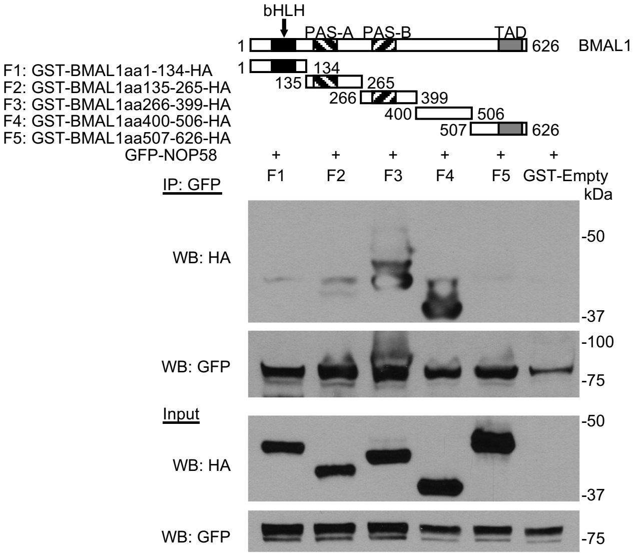

Figure S7 Related to Figure 5. BMAL1 PER-ARNT-SIM (PAS) domain is not required for NOP58 association

Schematic representation of BMAL1 showing the positions of the basic helix–loop–helix (bHLH), PER-ARNT-SIM (PAS)-A, PAS-B, and transactivation domain (TAD). GST-fused BMAL1-HA-tagged fragments are labeled as F1-F5 and are depicted with the range of amino acids (aa) for each peptide fragment. *in vitro* co-immunoprecipitation analyses were performed in whole cell extracts prepared from HEK293T cells co-transfected with GFP-NOP58 and incubated with GST-fused BMAL1 HA-tagged fragments or the GST-empty vector as indicated. Immunoprecipitation (IP) was performed using GFP followed by Western blot (WB) analyses of HA and GFP as specified.

TRANSPARENT METHODS

Animals

WT and *Bmal1*-KO mice were a generous gift from C. Bradfield (Bunger et al., 2000). Animals were housed under a temperature-controlled, 12-hr light/12-hr dark schedule, and fed *ad libitum*. Age-matched, male mice were sacrificed at specified zeitgeber times (ZT), livers were harvested, flash-frozen in liquid nitrogen and stored at -80ºC until use. All research involving vertebrate animals was performed in accordance with the guidelines of the Institutional Animal Care and Use Committee at the University of California, Irvine.

Cell culture

MEFs from *Bmal1*-KO, *Clock*Δ19 mutant mice and their respective WT were obtained as previously described (Aguilar-Arnal et al., 2013; Hirayama et al., 2007; Sahar et al., 2014), cultured in high-glucose DMEM (HyClone) supplemented with 10% (v/v) FBS (Gibco) and 1% (v/v) Pen/Strep (Gibco) antibiotics. HEK293T cells (ATCC) were maintained in high-glucose DMEM supplemented with 10% FBS and 1% antibiotics. HEK293T cells were transfected with indicated plasmids using BioT transfection reagent (Bioland Scientific LLC) according to manufacturer's recommendations. All cell lines were tested and free of mycoplasma contamination.

Plasmids

Myc-Bmal1 (Travnickova-Bendova et al., 2002), Myc-Clock (Doi et al., 2006), were previously described. GFP-Nop58 was a generous gift from E. Bertrand (Verheggen et al., 2002). Myc-Bmal1 was used as a template to generate Myc-Bmal1K538R and Myc-Bmal1K538Q point mutations using Q5 Site-Directed Mutagenesis (New England Biolabs) and NEBaseChanger to design mutagenesis primers. The primer sequences used were: Myc-Bmal1K538R: Fwd: 5’-TCCAGGAGGCcagAAGATTCTAAATG-3’, Rev: 5’-GAAGAGGCATCAGGGGGA-3’; Myc-

Bmal1K538Q: Fwd: 5’-TCCAGGAGGCaggAAGATTCTAAATG-3’, Rev: 5’-GAAGAGGCATCAGGGGGA-3’. RFP-Bmal1 was generated by subcloning *Bmal1* from Myc-Bmal1 into the EcoRI and BamHI sites of the pTagRFP-N vector (Evrogen). Control shRNA, pLKO.1-shGFP, was purchased from Addgene and shRNAs targeting *Bmal1* were generated using oligo sequences previously identified by The RNAi Consortium (Moffat et al., 2006) subcloned into the AgeI and EcoRI sites of pLKO.1-TRC (Addgene). The primer sequences used were renamed as: shBmal1 #1: Fwd: 5’-CCGGTCTTCAAGATCCTCAATTATACTCGAGTATAATTGAGGATCTTGAAGATTTTTG-3’, Rev: 5’-AATTCAAAAATCTTCAAGATCCTCAATTATACTCGAGTATAATTGAGGATCTTGAAGA-3’; shBmal1 #2: Fwd: 5’-CCGGGCAGTATCAAAGTGCATTAATCTCGAGATTAATGCACTTTGATACTGCTTTTTG-3’, Rev: 5’-AATTCAAAAAGCAGTATCAAAGTGCATTAATCTCGAGATTAATGCACTTTGATACTGC-3’; shBmal1 #3: Fwd: 5’-CCGGACATAGGCATCGATATGATAGCTCGAGCTATCATATCGATGCCTATGTTTTTTG-3’, Rev: 5’-AATTCAAAAAACATAGGCATCGATATGATAGCTCGAGCTATCATATCGATGCCTATGT-3’. Vectors for GST-Bmal1-HA fragments were generated by subcloning *Bmal1* sequences from cDNA encoding amino acids residues 1-134 for the F1, 135-265 for the F2, 266-399 for the F3, 400-506 for the F4, 507-626 for the F5 from mouse *Bmal1* (NCBI reference sequence: NM_007489.4) were cloned into the vector pGEX4T1 (GE Healthcare) with a TEV-cleavable N-terminal glutathione S-transferase (GST) tag and a C-terminal hemmaglutinin (HA) tag using EcoRI/NotI restriction sites. The primer sequences used were: GST-Bmal1aa1-134-HA: Fwd: 5’-TCCCCGGAATTCGAGAATCTTTATTTTCAGGGCATGGCGGACCAGAGAATGG-3’, Rev: 5’-CCACCAACCCATACACAGAATACCCTTATGATGTGCCGGATTATGCCTAGCGGCCGCATCG

TGAC; GST-Bmal1aa135-265-HA: Fwd: 5'-TCCCCGGAATTCGAGAATCTTTATTTTCAGGGCGCAAACTACAAGCCAACATTTC-3', Rev: 5'-AAAGGTGGAAGATAAGGACTTCTACCCTTATGATGTGCCGGATTATGCCTAGCGGCCGCATCGTGAC-3'; GST-Bmal1aa266-399-HA: Fwd: 5'-TCCCCGGAATTCGAGAATCTTTATTTTCAGGGCGCCTCTACCTGTAGAATGAAG-3', Rev: 5'-TTTTACAGACAAGAGAAAAGATCTACCCTTATGATGTGCCGGATTATGCCTAGCGGCCGCATCGTGAC-3'; GST-Bmal1aa400-506-HA: Fwd: 5'-TCCCCGGAATTCGAGAATCTTTATTTTCAGGGCACAACTAATTGCTATAAGTTTAAG-3', Rev: 5'-GCGGAGGAAATCATGGAAATCTACCCTTATGATGTGCCGGATTATGCCTAGCGGCCGCATCGTGAC-3'; GST-Bmal1aa507-626-HA: Fwd: 5'-TCCCCGGAATTCGAGAATCTTTATTTTCAGGGCCACAGGATAAGAGGGTCATC-3', Rev: 5'-GACTTGCCATGGCCGCTGTACCCTTATGATGTGCCGGATTATGCCTAGCGGCCGCATCGTGAC-3'. All the vectors were examined and verified by restriction site analysis and sequencing.

Lentiviral production and MEFs transduction

HEK293T cells were transfected with plasmids containing shRNAs, psPAX2, and VSV.G using BioT transfection reagent according to manufacturer's recommendations. After 16hrs, medium was replaced. Virus-containing media was filtered after 48hrs, supplemented with 8μg/ml Polybrene (Millipore) and added to WT MEFs for 16hrs. MEFs were selected for plasmid incorporation using 8μM puromyocin, overnight. Subsequent passages were maintained using growing media supplemented with 2μM puromyocin.

Immunofluorescence analysis

Cells were grown on Nunc Lab-Tek II chamber slides and transfected with plasmids as indicated. Frozen mouse liver tissues were cut to 10μm thick sections using Leica CM1950 Cryostat. Cells and tissue sections were fixed with ice cold 4% PFA for 20min at room temperature (RT) and washed three times with 1x PBS. Samples were then permeabilized (1x PBS, 0.3% Triton X-100) for 15min at RT and blocked (1x PBS, 5% BSA, 10% normal goat serum (NGS)) for 2hrs at RT. Following incubation with primary antibodies diluted in blocking buffer overnight at 4ºC, samples were washed three times with 1x PBS and incubated with secondary antibodies for 2hrs at RT. After three washes with 1x PBS, nuclei were stained using DRAQ7 (Biostatus) or DAPI (Life Technologies) for 15min at RT and subsequently washed twice with 1x PBS. Endogenous and ectopic proteins were detected using the following antibodies: BMAL1 (Novus, NB100-2288), NOP58 (Abcam, ab155969), FBL (single stain: CST, #2639; double stain: Novus, NB300-269), MYC (Millipore, 05-419). Secondary antibodies: Alexa Fluor 488 Goat anti-Rabbit IgG (Invitrogen, A-11008) and Alexa Fluor 546 Goat anti-Mouse IgG (Invitrogen, A-11003). Quantification of the total area and number of nucleoli were performed on immunofluorescence confocal images (157x157μm/image; Leica, SP5) of cells and liver tissue sections probed with NOP58 (Abcam, ab155969) or FBL (single stain: CST, #2639; double stain: Novus, NB300-269) using ImageJ software in the following sequence: Split channels > for the nuclear channel (DRAQ7 or DAPI): setAutoThreshold (Default dark, BlackBackground) > Convert to Mask > Fill Holes > Watershed > Analyze Particles (Size ≥ 200 pixels, Circularity = 0.00-1.00, Exclude on edges, Add to manager), for the nucleolar channel (NOP58 or FBL): setAutoThreshold (Default dark) > Convert to Mask > Watershed > Analyze Particles (Size ≥ 5 pixels, Circularity = 0.00-1.00, Exclude on edges, Show = Outlines) > overlay ROIs from manager > Measure (%Area).

Nucleolar fractionation

Nucleoli were prepared as previously described (Andersen et al., 2002) with minor modifications. Whole mouse livers were divided into 3 equal pieces and each minced in 5ml of Buffer A (10mM HEPES pH7.9, 1.5mM $MgCl_2$, 10mM KCl, 0.5mM DTT, supplemented with protease inhibitor cocktail (Roche), 0.5mM PMSF, 20mM NaF, 1μM TSA, 10mM NAM). Tissues were homogenized and spun at 1600rpm (600g) for 10min at 4ºC. Pellets were resuspended in 3ml of Buffer S1 (250mM Sucrose, 10mM $MgCl_2$, supplemented with protease inhibitor cocktail (Roche), 0.5mM PMSF, 20mM NaF, 1μM TSA, 10mM NAM), layered over 3ml of Buffer S2 (350mM Sucrose, 0.5mM $MgCl_2$, supplemented with protease inhibitor cocktail (Roche), 0.5mM PMSF, 20mM NaF, 1μM TSA, and 10mM NAM) and spun at 2500rpm (1430g) for 5min at 4ºC. All three nuclear pellets were combined using Buffer S2, sonicated, layered over 3ml of Buffer S3 (880mM Sucrose, 0.5mM $MgCl_2$, supplemented with protease inhibitor cocktail (Roche), 0.5mM PMSF, 20mM NaF, 1μM TSA, 10mM NAM) and spun at 3500rpm (2800g) for 10min at 4ºC. Supernatant was retained as the enriched nucleoplasmic fraction and diluted with 5x RIPA (250mM Tris pH7.5, 750mM NaCl, 5% NP-40, supplemented with protease inhibitor cocktail (Roche), 0.5mM PMSF, 20mM NaF, 1μM TSA, 10mM NAM). The nucleolar pellet was resuspended in 500μl of Buffer S2 and spun at 3500rpm (2800g) for 5min at 4ºC. The cleaned nucleolar pellet was resuspended in 1x RIPA (50mM Tris pH7.5, 150mM NaCl, 1% NP-40, supplemented with protease inhibitor cocktail (Roche), 0.5mM PMSF, 20mM NaF, 1μM TSA, 10mM NAM). Fractions were briefly sonicated, rocked for 30min at 4ºC, spun at 12500rpm (18000g) for 10min at 4ºC, and enriched lysates collected.

Western blot analysis

Whole cell lysates were prepared from mouse liver tissues by homogenization in 1x RIPA (50mM Tris pH7.5, 150mM NaCl, 5mM EDTA, 1% NP-40, 0.5% Deoxycholate, supplemented with protease inhibitor cocktail (Roche), 0.5mM PMSF, 20mM NaF, 1μM TSA, 10mM NAM), briefly sonicated, rocked for 30min at 4ºC, spun at 12500rpm (18000g) for 10min at 4ºC, and

lysates collected. Similarly, MEFs were harvested in 1x RIPA, sonicated, rocked and spun. 20μg of whole cell lysates or nucleolar fractionation lysates were resolved on 6%, 8%, or 10% SDS-PAGE. Antibodies used for Western blot include: BMAL1 (Abcam, ab93806), NOP58 (Abcam, ab155969), NOP56 (Proteintech, 18181-1-AP), FIBRILLARIN (CST, #2639), NUCLEOLIN (CST, #14574), Ribosomal Protein S6 (CST, #2217), p84 (Genetex, GTX70220), TUBULIN (Sigma, T5168). Secondary antibodies include: HRP-conjugate Goat anti-Rabbit IgG (Millipore, 12-348) and HRP-conjugate Rabbit anti-Mouse IgG (Millipore, AP160P).

RNA extraction, reverse transcription and quantitative real-time PCR analysis

Confluent WT and *Bmal1*-KO MEFs were synchronized by treatment with 100nM Dexamethasone (Sigma) for 30min as described previously (Aguilar-Arnal et al., 2013; Hirayama et al., 2007; Sahar et al., 2014). Total RNA, from either cells or WT and *Bmal1*-KO mouse livers collected at the indicated circadian times (CT or ZT, respectively), were extracted using TRIzol (Invitrogen) following manufacturer's recommendation. 1μg of total RNA was reverse-transcribed to cDNA using iScript cDNA synthesis kit (Bio-Rad Laboratories), according to manufacturer's protocol. cDNA (1:10) was used for quantitative real-time PCR using SsoAdvanced Universal SYBR Green Supermix (Bio-Rad Laboratories) and ran on a CFX96 Touch real-time PCR machine (Bio-Rad Laboratories). Gene expression was normalized to 18S rRNA (1:500 cDNA). Primer sequences used for gene expression analysis were designed with Primer3 software (Koressaar and Remm, 2007; Untergasser et al., 2012). 5'ETS/18S: Fwd: 5'-CTCCTCTCTCGCGCTCTCT-3', Rev: 5'-GGCCGTGCGTACTTAGACAT-3'; 18S/ITS1: Fwd: 5'-CTGAGAAGACGGTCGAACTTG-3', Rev: 5'-CCTCCACAGTCTCCCGTTTA-3'; ITS1/5.8S: Fwd: 5'-ACACCCGAAATACCGATACG-3', Rev: 5'-GTGCGTTCGAAGTGTCGAT-3'; ITS2/28S: Fwd: 5'-GCCTCCTCGCTCTCTTCTTC-3', Rev: 5'-GCCGTTACTGAGGGAATCCT-3'; Rnu3a (U3): Fwd: 5'-ACTGTGTAGAGCACCCGAAAC-3', Rev: 5'-GACTGTGTCCTCTCCCTCTCA-3'; Snord118 (U8): Fwd: 5'-CCTTACCTGTTCCTCCTTTCG-3', Rev: 5'-

GAGCAACCAGGATGTTGTCA-3'; Snord17: Fwd: 5'-TGACCTTCTTCCCAGTCTCG-3', Rev: 5'-GGTGAGATGGAACCCAGAGA-3'; 18S: Fwd: 5'-CGCCGCTAGAGGTGAAATTC-3', Rev: 5'-CGAACCTCCGACTTTCGTTCT-3'.

Northern blot analysis

Total RNA was extracted from WT and *Bmal1*-KO MEFs using TRIzol (Invitrogen) following manufacturer's recommendation. 20µg of total RNA was equally added to 1x TT (1.5M Triethanolamine, 1.5M Tricine), 1mM EDTA, 6.67% Formaldehyde, 50% Formamide, and incubated at 65ºC for 15min. Samples were then mixed with 6x Formaldehyde loading buffer (1mM EDTA, 0.25% Bromophenol blue, 50% Glycerol) and ran on a 0.8% Agarose-Formaldehyde gel (1x TT, 6.67% Formaldehyde) at 4ºC for 15hrs. The gel was prepared for transfer by soaking in 0.05N NaOH for 20min and in 20x SSC (3M NaCl, 0.3M Sodium citrate) for 45min at RT. RNA was transferred overnight onto a positively charged nylon membrane (Amersham Biosciences) by capillary transfer in 10x SSC at RT. The membrane was then rinsed in 2x SSC and UV crosslinked for 15min, incubated in Prehybridization buffer (5x SSC, 5x Denhardt's solution, 0.5% SDS, 25µg/ml Salmon sperm) for 2hrs at 65ºC and hybridized overnight at 65ºC using previously identified probes (Lapik et al., 2004) diluted in prehybridization buffer. After three washes in Wash buffer (2x SSC, 0.1% SDS), the membrane was dried, exposed to a BAS-MS FUJI imaging plate (FUJIFILM) and imaged using Amersham Typhoon imager (GE Healthcare). Probes were labeled at 37ºC for 1hr in a mixture of 1µM Oligo, 1x T4 PNK reaction buffer (New England Biolabs), 20U T4 PNK (New England Biolabs), 50µCi [γ-^{32}P] ATP 3,000Ci/mmol (PerkinElmer). Labeled probes were purified using illustra Microspin G-25 columns (GE Healthcare) following manufacturer's instructions. Probe sequences used were previously identified by (Lapik et al., 2004); probes were labeled as Pink probe (5'ETS): 5'-AGCTCCCCACGGGAAAGCAATGAGTCTCTC-3'; Gold probe (ITS2): 5'-ACCCACCGCAGCGGGTGACGCGATTGATCG-3'; Blue probe (ITS1): 5'-

CTCTCACCTCACTCCAGACACCTCGCTCCA-3'. Imaged blots were quantitated by densitometry analysis on ImageJ software, pre-rRNA intermediates were normalized to 47S/45S. Ratio Analysis of Multiple Precursors (RAMP) analysis was performed as previously described (Wang et al., 2014).

Co-immunoprecipitation

1-4mg of protein were prepared from whole cell lysates of transfected cells or nucleolar fractions from mouse liver tissues in 1x RIPA (50mM Tris pH7.5, 150mM NaCl, 1% NP-40, supplemented with protease inhibitor cocktail (Roche), 0.5mM PMSF, 20mM NaF, 1μM TSA, 10mM NAM). Lysates were pre-cleared with 20μl of Protein G Sepharose beads (Sigma) while rocking at 4ºC for 1hr. After removal of pre-clearing beads, 10% of lysate was retained as input, the remainder of lysates were then incubated with 4μg of BMAL1 antibody (Abcam, ab93806), 4μg of NOP58 antibody (Proteintech, 14409-1-AP), or 5μg of GFP antibody (Abcam, ab290) as indicated. An additional, pooled sample was prepared with equal amount of protein for incubation with equal amount of Rabbit IgG (Santa Cruz Biotechnology, sc-2027 or Invitrogen, 10500C), and rocked overnight at 4ºC. The next day, 40μl of fresh Protein G Sepharose beads were added to the lysates and rocked at 4ºC for 2hrs. Beads were washed three times with 1x RIPA. 50μl sample buffer (240mM Tris-HCl pH 6.8, 40% Glycerol, 8% SDS, 20% β-mercaptoethanol, 0.02% Bromophenol blue) was added directly to the beads and samples were boiled for 10min at 95ºC. 20μl of samples and input were analyzed by Western blot. Antibodies used for Western blot include: BMAL1 (Abcam, ab93806), NOP58 (Abcam, ab155969), FIBRILLARIN (CST, #2639), SIRT7 (SCBT, sc-365344), AcBMAL1 (Millipore, 15396), MYC (Millipore, 05-419), GFP (Abcam, ab6556). Secondary antibodies include: HRP-conjugate Mouse anti-Rabbit light chain (Millipore, MAB201P) and HRP-conjugate Rabbit anti-Mouse IgG (Millipore, AP160P).

***in vitro* co-immunoprecipitation**

GST-fused recombinant BMAL1-HA fragments and GST-empty vectors were expressed in *Escherichia coli* and purified as described in detail previously (Delvecchio et al., 2013). Briefly, fusion proteins were expressed in *E.coli*, BL21 (DE3) and purified using glutathione Sepharose 4 Fast Flow resin according to the manufacturer protocol (GE Healthcare). 1μg of each GST-purified recombinant BMAL1 HA-tagged fragments were incubated overnight at 4ºC while rocking with 1mg of whole cell extracts from HEK293T cells transfected with GFP-NOP58. Lysates were then pre-cleared with 20μl of Protein G Sepharose beads (Sigma) while rocking at 4ºC for 1hr. After removal of pre-clearing beads, 10% of lysate was retained as input, the remainder of lysates were then incubated with 5μg of GFP antibody (Abcam, ab290) and rocked overnight at 4ºC. The next day, 40μl of fresh Protein G Sepharose beads were added to the lysates and rocked at 4ºC for 2hrs. Beads were washed three times with 1x RIPA. 50μl sample buffer (240mM Tris-HCl pH 6.8, 40% Glycerol, 8% SDS, 20% β-mercaptoethanol, 0.02% Bromophenol blue) was added directly to the beads and samples were boiled for 10min at 95ºC. 20μl of samples and input were analyzed by Western blot. Antibodies used for Western blot include: HA (Millipore, 05-904) and GFP (Abcam, ab6556). Secondary antibodies include: HRP-conjugate Mouse anti-Rabbit light chain (Millipore, MAB201P) and HRP-conjugate Rabbit anti-Mouse IgG (Millipore, AP160P).

Liquid chromatography-mass spectrometry

2mg of nucleoplasmic and nucleolar fractions from WT mouse livers harvested at ZT8 and ZT20 were prepared in 1x RIPA (50mM Tris pH7.5, 150mM NaCl, 1% NP-40, supplemented with protease inhibitor cocktail (Roche), 0.5mM PMSF, 20mM NaF, 1μM TSA, 10mM NAM) and 4μg of BMAL1 antibody (Abcam, ab93806) was used for co-immunoprecipitation as described above; after the final wash with 1x RIPA, the beads were washed three times with 50mM NH4HCO3 and incubated with 10 ng/μL trypsin in 1 M urea 50mM NH4HCO3 for 30 minutes, washed with 50mM NH4HCO3 and the supernatant digested overnight (ON) in presence of

1mM DTT. Digested peptides were alkylated and desalted prior to LC-MS analysis. For LC-MS/MS purposes, desalted peptides were injected in an Ultimate 3000 RSLCnano system (Thermo), separated in a 15-cm analytical column (75μm ID home-packed with ReproSil-Pur C18-AQ 2.4 μm from Dr. Maisch) with a 50-min gradient from 5 to 60% acetonitrile in 0.1% formic acid. The effluent from the HPLC was directly electrosprayed into a Qexactive HF (Thermo) operated in data dependent mode to automatically switch between full scan MS and MS/MS acquisition. Survey full scan MS spectra (from m/z 375–1600) were acquired with resolution R=60,000 at m/z 400 (AGC target of 3x106). The 10 most intense peptide ions with charge states between 2 and 5 were sequentially isolated to a target value of 1x105, and fragmented at 27% normalized collision energy. Typical mass spectrometric conditions were: spray voltage, 1.5 kV; no sheath and auxiliary gas flow; heated capillary temperature, 250ºC; ion selection threshold, 33.000 counts. MaxQuant 1.5.2.8 was used to identify proteins and quantify by iBAQ with the following parameters: Database, UP000000589_10090_Mmusculus_151030; MS tol, 10ppm; MS/MS tol, 0.5 Da; Peptide FDR, 0.1; Protein FDR, 0.01 Min. peptide Length, 5; Variable modifications, Oxidation (M); Fixed modifications, Carbamidomethyl (C); Peptides for protein quantitation, razor and unique; Min. peptides, 1; Min. ratio count, 2. Identified proteins were considered nucleolar-enriched interaction partners if they met both requirements: their MaxQuant iBAQ values displayed greater than two-fold enrichment with $p<0.05$ (two-way ANOVA adjusted for multiple comparisons) when compared to the IgG control and if their MaxQuant iBAQ values displayed greater than four-fold enrichment with $p<0.05$ (two-way ANOVA adjusted for multiple comparisons) when compared to the nucleoplasmic interaction partners. Gene Ontology (GO) analysis was performed on the identified nucleolar-enriched interaction partners using the Database for Annotation, Visualization and Integrated Discovery (DAVID) v6.8 (Huang da et al., 2009). MS data are available via ProteomeXchange with identifier PXD018946.

Fluorescence lifetime imaging

HEK293T cells were plated in 35-mm glass-bottom microwell dishes (MatTek) and transfected with GFP-NOP58 alone or GFP-NOP58 and RFP-BMAL1. After 24hr, FLIM images of the two cell types - donor only (GFP-NOP58) and donor-acceptor (GFP-NOP58:RFP-BMAL1) were measured using a modified Olympus Fluoview FV1000 (Olympus, Waltham, MA) microscope equipped with a Spectra-Physics MaiTai HP laser (Spectra Physics, Santa Clara, CA) and FLIMBox (ISS, Champaign, IL) acquisition card. 900 nm laser line using a 63X water immersion objective (1.2 NA, Olympus Plan-apo, Olympus, Watltham, MA) were used for excitation applying a two photon excitation scheme. The resulting fluorescence was collected using the same objective and was split in two channels using a dichroic mirror (FF495-Di03-25x36, Semrock, Rochester, NY) and then passed through two separate filters for GFP (520/35 nm, Semrock) and RFP (641/75 nm, Semrock) channels, and collected using two separate photomultiplier tube (H7422P-40, Hamamatsu, Bridgewater, NJ), and recorded using FLIMBox. The pixel dwell time for the acquisitions was set at 32 µs and the images were taken with sizes of 256x256 pixels. To have high signal to noise ratio, 20 – 30 frames were collected. Scanning and field of view were controlled by Olympus software and the data were acquired in passive FLIMBox mode. The data from each pixel were recorded and analyzed using the SimFCS software (developed by Dr. Enrico Gratton in Laboratory for Fluorescence Dynamics, University of California, Irvine, CA). The intensity decays collected at each pixel of the image were transformed to the Fourier space and the phasor coordinates were calculated using the following relations:

$$g_{i,j}(\omega) = \int_0^T I(t) \cdot Cos(n\omega t)dt \Big/ \int_0^T I(t)dt$$

$$s_{i,j}(\omega) = \int_0^T I(t) \cdot Sin(n\omega t)dt \Big/ \int_0^T I(t)dt$$

where, $g_{i,j}(\omega)$ and $s_{i,j}(\omega)$ are the X and Y coordinates of the phasor plot, respectively, and n and ω are the harmonic number and the angular frequency of excitation, respectively, and T is the repeat frequency of laser (80 MHz).

RNA immunoprecipitation followed by quantitative PCR (RIP-qPCR)

1.5mg of nucleolar fractions from WT and *Bmal1*-KO mouse livers harvested at ZT8 were prepared in 1x RIP buffer (25mM Tris pH7.5, 150mM KCl, 5mM EDTA, 0.5mM DTT, 0.5% NP-40, supplemented with protease inhibitor cocktail (Roche), 0.5mM PMSF, 20mM NaF, 1μM TSA, 10mM NAM, 100U/ml RNaseOUT (Invitrogen)) and 4μg of NOP58 antibody (Proteintech, 14409-1-AP) was used for co-immunoprecipitation as described above with a modification to buffers, S2 and S3, the addition of 100U/ml RNaseOUT (Invitrogen). After the final wash with 1x RIP buffer, 1ml of TRIzol was added to the beads and input samples and RNA was isolated following manufacturer's recommendation. RNA was resuspended in equal volume of DEPC-treated water and reversed transcribed into cDNA using Maxima H Minus cDNA Synthesis Master Mix (Thermo Scientific, Cat#1661). cDNA was used for quantitative real-time PCR using SsoAdvanced Universal SYBR Green Supermix (Bio-Rad Laboratories) and ran on a QuantStudio 3 real-time PCR System (Applied Biosystems). Primer sequences used to identify bound-RNA were designed with Primer3 software (Koressaar and Remm, 2007; Untergasser et al., 2012). Rnu3a (U3): Fwd: 5'-ACTGTGTAGAGCACCCGAAAC-3', Rev: 5'-GACTGTGTCCTCTCCCTCTCA-3'; Snord118 (U8): Fwd: 5'-CCTTACCTGTTCCTCCTTTCG-3', Rev: 5'-GAGCAACCAGGATGTTGTCA-3'; Snord17: Fwd: 5'-TGACCTTCTTCCCAGTCTCG-3', Rev: 5'-GGTGAGATGGAACCCAGAGA-3'; 18S: Fwd: 5'-CGCCGCTAGAGGTGAAATTC-3', Rev: 5'-CGAACCTCCGACTTTCGTTCT-3'; 5'ETS/18S: Fwd: 5'-CTCCTCTCTCGCGCTCTCT-3', Rev: 5'-GGCCGTGCGTACTTAGACAT-3'; 18S/ITS1: Fwd: 5'-CTGAGAAGACGGTCGAACTTG-3', Rev: 5'-CCTCCACAGTCTCCCGTTTA-3'; ITS1/5.8S: Fwd: 5'-ACACCCGAAATACCGATACG-3', Rev: 5'-GTGCGTTCGAAGTGTCGAT-3'; ITS2/28S: Fwd:

5'-GCCTCCTCGCTCTCTTCTTC-3', Rev: 5'-GCCGTTACTGAGGGAATCCT-3'; Actin: Fwd: 5'-GGCTGTATTCCCCTCCATG-3', Rev: 5'-CCAGTTGGTAACAATGCCATGT-3'.

Polysome profiling

WT and *Bmal1*-KO MEFs were treated with 10µg/ml Cycloheximide on a rocker for 5min at RT. One plate of each WT and *Bmal1*-KO MEF cells was retained for standard DNA extraction by Phenol-chloroform. The remainder of cells were washed with 1x PBS containing 200µg/ml of cycloheximide, scraped to collect, and spun at 1500rpm (500g) for 5min. Cell pellets were homogenized in equal volume of Homogenization buffer (300mM NaCl, 50mM Tris-HCl pH8.0, 10mM $MgCl_2$, 1mM EGTA, 1% Triton X-100, 0.1% DOC, 200µg/ml Heparin, 1mM DTT, 200U/ml RNaseOUT (Invitrogen), supplemented with protease inhibitor cocktail (Roche), 200µg/ml Cycloheximide), rocked at 4ºC for 10min, and spun at 12500rpm (18000g) for 15min at 4ºC. Each polysomal lysate was normalized to DNA concentration and layered above a 10-50% sucrose gradient (140mM NaCl, 25mM Tris-HCl pH8.0, 10mM $MgCl_2$, 10%-50% Sucrose) and centrifuged at 31000rpm for 2hrs at 4ºC. Gradients were run through a UA-6 UV/Vis detector (Teledyne Isco) to record polysome profiles. Graphs were digitalized using WebPlotDigitizer software (https://automeris.io/WebPlotDigitizer).

Statistical analysis

Sample size and data presented as mean + s.d. or plotted as individual data points were indicated in the figure legends. Statistical significance was determined by t test, one-way ANOVA, or two-way ANOVA (GraphPad Prism8) as indicated in the figure legends. Statistical significance was assigned as *, **, ***, and **** when p-value cutoffs of 0.05, 0.01, 0.001, and 0.0001, respectively, were met.

SUPPLEMENTAL REFERENCES

Aguilar-Arnal, L., Hakim, O., Patel, V.R., Baldi, P., Hager, G.L., and Sassone-Corsi, P. (2013). Cycles in spatial and temporal chromosomal organization driven by the circadian clock. Nat Struct Mol Biol *20*, 1206-1213.

Andersen, J.S., Lyon, C.E., Fox, A.H., Leung, A.K., Lam, Y.W., Steen, H., Mann, M., and Lamond, A.I. (2002). Directed proteomic analysis of the human nucleolus. Curr Biol *12*, 1-11.

Bunger, M.K., Wilsbacher, L.D., Moran, S.M., Clendenin, C., Radcliffe, L.A., Hogenesch, J.B., Simon, M.C., Takahashi, J.S., and Bradfield, C.A. (2000). Mop3 is an essential component of the master circadian pacemaker in mammals. Cell *103*, 1009-1017.

Delvecchio, M., Gaucher, J., Aguilar-Gurrieri, C., Ortega, E., and Panne, D. (2013). Structure of the p300 catalytic core and implications for chromatin targeting and HAT regulation. Nat Struct Mol Biol *20*, 1040-1046.

Doi, M., Hirayama, J., and Sassone-Corsi, P. (2006). Circadian regulator CLOCK is a histone acetyltransferase. Cell *125*, 497-508.

Henras, A.K., Plisson-Chastang, C., O'Donohue, M.F., Chakraborty, A., and Gleizes, P.E. (2015). An overview of pre-ribosomal RNA processing in eukaryotes. Wiley Interdiscip Rev RNA *6*, 225-242.

Hirayama, J., Sahar, S., Grimaldi, B., Tamaru, T., Takamatsu, K., Nakahata, Y., and Sassone-Corsi, P. (2007). CLOCK-mediated acetylation of BMAL1 controls circadian function. Nature *450*, 1086-1090.

Huang da, W., Sherman, B.T., and Lempicki, R.A. (2009). Systematic and integrative analysis of large gene lists using DAVID bioinformatics resources. Nat Protoc *4*, 44-57.

Koressaar, T., and Remm, M. (2007). Enhancements and modifications of primer design program Primer3. Bioinformatics *23*, 1289-1291.

Lapik, Y.R., Fernandes, C.J., Lau, L.F., and Pestov, D.G. (2004). Physical and functional interaction between Pes1 and Bop1 in mammalian ribosome biogenesis. Molecular cell *15*, 17-29.

Moffat, J., Grueneberg, D.A., Yang, X., Kim, S.Y., Kloepfer, A.M., Hinkle, G., Piqani, B., Eisenhaure, T.M., Luo, B., Grenier, J.K.*, et al.* (2006). A lentiviral RNAi library for human and mouse genes applied to an arrayed viral high-content screen. Cell *124*, 1283-1298.

Sahar, S., Masubuchi, S., Eckel-Mahan, K., Vollmer, S., Galla, L., Ceglia, N., Masri, S., Barth, T.K., Grimaldi, B., Oluyemi, O.*, et al.* (2014). Circadian control of fatty acid elongation by SIRT1 protein-mediated deacetylation of acetyl-coenzyme A synthetase 1. The Journal of biological chemistry *289*, 6091-6097.

Travnickova-Bendova, Z., Cermakian, N., Reppert, S.M., and Sassone-Corsi, P. (2002). Bimodal regulation of mPeriod promoters by CREB-dependent signaling and CLOCK/BMAL1 activity. Proceedings of the National Academy of Sciences of the United States of America *99*, 7728-7733.

Untergasser, A., Cutcutache, I., Koressaar, T., Ye, J., Faircloth, B.C., Remm, M., and Rozen, S.G. (2012). Primer3--new capabilities and interfaces. Nucleic acids research *40*, e115.

Verheggen, C., Lafontaine, D.L., Samarsky, D., Mouaikel, J., Blanchard, J.M., Bordonne, R., and Bertrand, E. (2002). Mammalian and yeast U3 snoRNPs are matured in specific and related nuclear compartments. EMBO J *21*, 2736-2745.

Wang, M., Anikin, L., and Pestov, D.G. (2014). Two orthogonal cleavages separate subunit RNAs in mouse ribosome biogenesis. Nucleic acids research *42*, 11180-11191.
